# AI-Assisted Multilabel Diagnosis of 12-Lead Electrocardiograms Using an Interpretable Stacked Deep Learning Model with External Validation

**DOI:** 10.3390/diagnostics16172781

**Published:** 2026-08-29

**Authors:** Asifa Tassaddiq, Aiman Albarakati, Rabab Alharbi, Carlo Cattani, Dalal Khalid Almutairi, Ruhaila Md Kasmani

**Affiliations:** 1 Department of Information Technology, College of Computer and Information Sciences, Majmaah University, Al Majmaah 11952, Saudi Arabia; a.tassaddiq@mu.edu.sa; 2 Department of Computer Engineering, College of Computer and Information Sciences, Majmaah University, Al Majmaah 11952, Saudi Arabia; 3 Department of Mathematics, College of Science, Qassim University, Buraydah 51452, Saudi Arabia; ras.alharbi@qu.edu.sa; 4 Engineering School (DEIM), University of Tuscia, 01100 Viterbo, Italy; cattani@unitus.it; 5 Department of Mathematics, College of Science, Al Zulfi, Majmaah University, Al Majmaah 11952, Saudi Arabia; 6 Institute of Mathematical Sciences, Universiti Malaya, Kuala Lumpur 50603, Malaysia; ruhaila@um.edu.my

**Keywords:** ECG diagnosis, AI-assisted diagnosis, clinical decision support, multi-label classification, external validation, artificial intelligence, PTB-XL, Ningbo, InceptionTime, Grad-CAM

## Abstract

**Background:** Automated interpretation of 12-lead electrocardiograms (ECGs) remains challenging because multiple abnormalities may coexist and appear in selected leads or brief waveform segments. We developed a compact and interpretable framework for five-superclass multi-label ECG diagnosis. **Methods:** We evaluated PTB-XL records using the official fold protocol, with folds 1–8 for training, fold 9 for validation monitoring and class-specific threshold selection, and fold 10 for independent internal testing. We then evaluated the frozen 1.33-million-parameter InceptionTime–CNN–BiGRU–Transformer model and validation-derived thresholds on 15,931 Ningbo ECGs without retraining, recalibration, or external threshold adjustment. We also examined calibration, demographic subgroups, computational efficiency, and complementary ECG-domain attribution methods. **Results:** Macro-AUROC reached 90.83% on PTB-XL fold 10 and 88.85% on Ningbo, indicating generally consistent diagnostic ranking with a modest reduction during external evaluation. Sensitivity analysis showed that CNN-only outperformed the frozen primary model on five of six endpoints. Attribution analyses highlighted qualitatively plausible lead and temporal patterns in representative examples, while calibration and subgroup analyses further characterized model behavior under dataset shift. **Conclusions:** Our framework integrates leakage-aware development, threshold-controlled testing, frozen external validation, and multimethod interpretability. These findings support its further prospective, locally calibrated evaluation as a potential aid for multi-label ECG interpretation.

## 1. Introduction and Motivation

### 1.1. Motivation

Cardiovascular diseases remain a leading cause of morbidity and mortality worldwide. Technical standardization is essential for reliable 12-lead electrocardiogram (ECG) acquisition and interpretation [1]. Standardized criteria also support consistent reporting of intraventricular conduction disturbances [2]. In real clinical practice, ECG abnormalities often co-exist; myocardial infarction (MI), ST/T changes, conduction disturbance (CD), and hypertrophy (HYP) may appear in the same recording. Motivated by these facts, we consider five-superclass ECG diagnosis as a multi-label classification task rather than as a single-class problem [3]. By doing so, we evaluate a compact InceptionTime–CNN–BiGRU–Transformer framework for the five clinically interpretable PTB-XL diagnostic superclasses: normal ECG (NORM), MI, ST/T change (STTC), CD, and HYP. Unlike studies that rely on random splits, selected subclasses, non-official partitions, or test-dependent thresholds, this work uses the official PTB-XL fold protocol, validation-derived class-specific thresholds, and frozen external validation on the independent Ningbo cohort without retraining, recalibration, or post hoc threshold adjustment. This conservative design is in line with guidance for medical-AI assessment emphasizing transparent reporting, split-before-preprocessing practice, and avoidance of data leakage [4]. It is also consistent with recommendations that responsible medical-AI evaluation encompass external validation, robustness, and reliability rather than internal accuracy alone [5]. We examine model behavior using ECG-domain audit methods, including lead occlusion, temporal occlusion, attention profiling, Grad-CAM, and GradientSHAP. Our purpose is to estimate the discrimination, fixed-threshold transfer, and failure modes of a compact five-superclass ECG-AI research model on an independent external source.

### 1.2. Review of Literature

Deep learning has substantially advanced automated ECG interpretation by learning diagnostic representations directly from waveform data. Large-scale neural networks have achieved cardiologist-level arrhythmia detection from ambulatory ECG recordings [6]. Deep learning has also been used for large-scale automatic diagnosis from 12-lead ECG signals [7]. AI-enabled ECG screening has further been extended to clinically relevant tasks such as detection of cardiac contractile dysfunction [8]. Benchmarking work on PTB-XL has emphasized that ECG-AI performance should be assessed using standardized datasets and reproducible evaluation protocols [9].

Several deep learning architectures provide the technical foundation for modern ECG representation learning. Convolutional neural networks are effective for extracting local waveform morphology and have become widely used in time-series classification [10]. InceptionTime-style architectures extend this idea by using multiple temporal receptive fields to capture short- and long-duration signal patterns [11]. Long short-term memory networks provide a classical recurrent approach for modeling temporal dependencies [12]. Gated recurrent units offer a related recurrent mechanism for sequential representation learning with fewer gating components [13]. Attention mechanisms allow a model to emphasize informative parts of a sequence during prediction [14]. Transformer encoders further generalize attention-based modeling and are useful for capturing longer-range temporal interactions [15]. Residual learning has also supported the training of deeper feature extractors while preserving useful signal representations [16].

Reliable ECG-AI evaluation requires careful dataset design and external validation. PTB-XL provides a large public 12-lead ECG resource with waveform data, diagnostic statements, superclass labels, metadata, and recommended folds [17,18]. More broadly, PhysioNet has provided essential infrastructure for reproducible physiological-signal research [19]. The PhysioNet/Computing in Cardiology Challenge demonstrated that heterogeneous 12-lead ECG sources introduce substantial variation in labels, recording conditions, and patient populations [20]. Its Ningbo partition provides a large independent source for external transfer evaluation [21]. Recent multi-source ECG work has shown that conventional internal validation can overestimate performance when the intended setting involves unseen clinical sources [22].

Interpretability is equally important because a high performance score does not confirm that the model used clinically plausible ECG evidence. LIME provides local surrogate explanations for individual model predictions [23]. SHAP provides an additive feature-attribution framework for explaining model outputs [24]. Grad-CAM provides gradient-based localization of discriminative regions in deep neural networks [25]. Broader explainable-AI research emphasizes the need to make deep learning decisions more transparent, interpretable, and auditable [26]. Recent interpretable multi-label ECG work has shown the value of combining attention-enhanced deep learning with SHAP and Grad-CAM explanations [27].

Recent hybrid and clinically structured ECG-AI studies further motivate the present design. CNN–Transformer and hybrid 12-lead networks combine local feature extraction with temporal-context modeling [28,29]. Lead-wise multi-branch models use complementary anatomical views for multi-label classification [30], while xECGArch separates shorter morphology from longer rhythm-related features [31]. Natural-language-supervised ECG representation learning has broadened the move toward generalizable clinical representations [32]. Most recently, Al-Bairmani et al. reported a compact CNN–BiLSTM–Transformer with SHAP explanations for a 27-label PhysioNet/CinC task [33]. That study is a relevant architectural comparator, but its label space, source mixture, split, and headline metrics differ from the present five-superclass official-fold and frozen-Ningbo protocol; numerical superiority cannot be inferred from unpaired cross-study scores.

External ECG evaluation also requires careful dataset harmonization and transparent reporting. The Ningbo partition released through PhysioNet contains 34,905 12-lead ECGs and is distinct from the earlier 10,646-record Chapman/Shaoxing cohort [21,34]. The later combined PhysioNet database documents 500-Hz, 10 s recordings acquired with a GE MUSE system, and professional diagnostic labels [35]. Heterogeneous labels and populations require conservative mapping when transferring models across datasets [36].

Rare-label imbalance affects both training and evaluation. In the PTB-XL training folds, HYP had 2119 positive records compared with 7596 for NORM; in Ningbo, HYP and MI represented only 637 and 880 positive records, respectively. We therefore used positive-class weighting during training, validation-only class-specific thresholds, macro-averaged metrics, and AUPRC alongside AUROC. These measures reduce dominance by frequent labels but do not remove the limited morphological diversity or calibration uncertainty of rare labels. More targeted strategies—for example, asymmetric or focal objectives, balanced mini-batches, clinically reviewed augmentation, and hierarchical label modeling—remain important future work [37]. Clinical-AI prediction models should also follow transparent reporting guidance such as TRIPOD+AI, while calibration and future prospective studies require dedicated assessment [38,39,40].

Overall, prior work provides strong foundations in ECG deep learning, time-series architecture design, external validation, explainability, calibration, and clinical-AI reporting. However, important gaps remain for clinically oriented multi-label ECG diagnosis: some studies use non-official or random splits, some rely mainly on internal accuracy, some tune thresholds too close to the test setting, and many explanations are not reported in ECG-domain units. We address these gaps by evaluating a prespecified InceptionTime–CNN–BiGRU–Transformer model for five PTB-XL diagnostic superclasses. We combine official-fold development, validation-derived class-specific thresholds, frozen Ningbo external validation, calibration and reliability analysis, and lead-level and temporal interpretability through occlusion, attention, Grad-CAM, and GradientSHAP. Our main contributions are summarized as follows:

1. We develop a leakage-safe multi-label ECG workflow using the official PTB-XL folds: folds 1–8 for training, fold 9 for validation and threshold tuning, and fold 10 for independent internal testing.
2. We evaluate a compact stacked InceptionTime–CNN–BiGRU–Transformer model for five-superclass ECG diagnosis, combining multi-scale morphology learning, sequence modeling, Transformer context encoding, and temporal attention pooling.
3. We conduct two complementary post hoc analyses: a protocol-matched exact-parent ablation to assess component effects within the primary architecture and an architecture sensitivity analysis to examine performance across separately parameterized alternatives.
4. We select class-specific thresholds on the PTB-XL validation fold and freeze them before internal testing and external Ningbo evaluation, thereby avoiding test-set or external-cohort threshold optimization.
5. We evaluate the prespecified PTB-XL-trained checkpoint once on 15,931 eligible Ningbo ECGs without external retraining, recalibration, threshold optimization, candidate comparison, or post hoc decision adjustment.
6. We report ECG-level bootstrap confidence intervals, per-class ECE/Brier scores, and corrected descriptive age/sex subgroup analyses.
7. We provide ECG-domain interpretability through representative multi-label ECG panels, lead occlusion, temporal occlusion, attention profiling, external Ningbo Grad-CAM temporal localization, and supplementary GradientSHAP lead-attribution analysis.
8. We describe the clinical cohorts and report class-wise results and error analyses to clarify how performance differs across diagnostic superclasses and under external source shift.

The remainder of this paper is organized as follows. Section 2 describes the study design, clinical target task, PTB-XL and Ningbo cohorts, leakage-control protocol, and descriptive cohort characteristics. Section 3 presents the proposed framework, preprocessing, threshold-tuning procedure, and interpretability design. Section 4 describes the training protocol, post hoc architectural analyses, and evaluation metrics. Section 5 reports the internal and external validation results. Section 6 discusses the findings, comparisons with previous studies, limitations, and future directions.

## 2. Materials and Methods

### 2.1. Study Design and Clinical Target

We designed this study as a retrospective, externally validated, multi-label ECG-AI investigation. We deliberately restricted the diagnostic target to the five PTB-XL diagnostic superclasses: normal ECG (NORM), myocardial infarction (MI), ST/T change (STTC), conduction disturbance (CD), and hypertrophy (HYP). We selected these superclasses because they represent clinically interpretable ECG categories and can be conservatively mapped between PTB-XL and the independent Ningbo cohort.

We developed the model using PTB-XL and tested it externally on Ningbo. Following the official fold protocol, we used PTB-XL for training, validation, threshold selection, and internal testing. We accessed Ningbo only after fixing the final checkpoint and validation-derived class-specific thresholds, and we did not use any Ningbo record for training, hyperparameter selection, calibration fitting, threshold optimization, or post hoc decision adjustment. This design allowed us to evaluate whether a PTB-XL-trained five-superclass ECG model could retain diagnostic discrimination under an independent external source shift.

### 2.2. Dataset Flow and Leakage-Control Protocol

**Figure 1:**
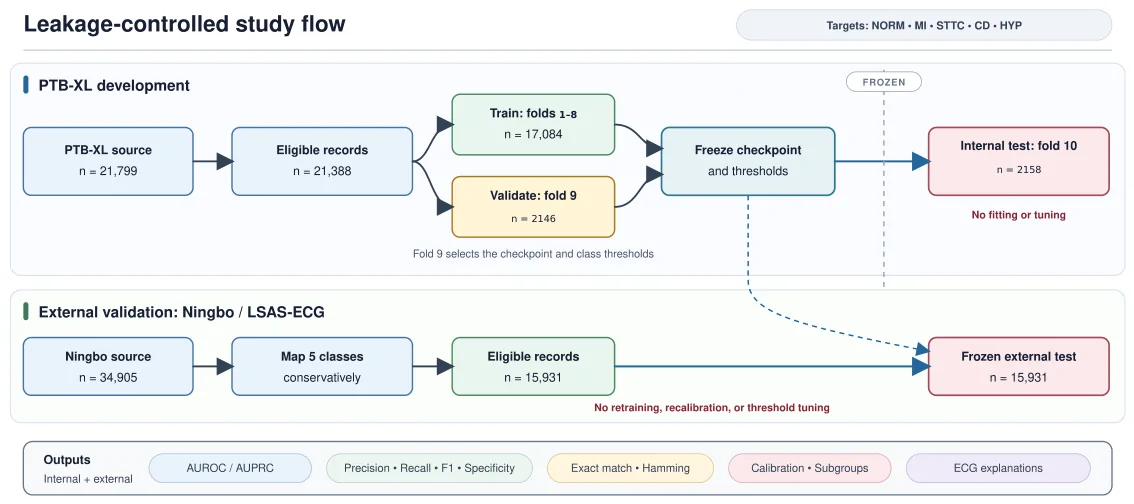
Dataset flow and leakage-control design.

Figure 1 summarizes our dataset flow and leakage-control design. We used PTB-XL folds 1–8 for model training and fold 9 for validation monitoring and class-specific threshold selection, while reserving fold 10 for independent internal testing. We evaluated the Ningbo cohort only after freezing the model checkpoint and all thresholds. This separation ensured that neither the internal test fold nor the external cohort influenced model fitting or decision-rule selection.

### 2.3. PTB-XL Internal Development and Test Cohort

We used the PTB-XL database as the internal source for model development and testing [17]. The public PTB-XL release provides waveform recordings, metadata, diagnostic statements, superclass labels, and recommended folds for reproducible evaluation [18]. The metadata contained 21,799 ECG records. We excluded records without at least one valid diagnostic superclass label, leaving 21,388 eligible ECGs for the five-superclass multi-label task.

The five target superclasses were NORM, MI, STTC, CD, and HYP. These labels are not mutually exclusive; therefore, one ECG may contain more than one diagnostic superclass. This clinical property motivated the use of five independent sigmoid outputs rather than a softmax classifier. Table 1 summarizes the clinical meaning, common ECG evidence, and interpretation caveat for each superclass.

The official PTB-XL split produced 17,084 training records, 2146 validation records, and 2158 test records. We did not use the test fold during model fitting, hyperparameter selection, threshold optimization, calibration, or any other tuning step. We adopted this protocol to reduce information leakage and make the internal evaluation reproducible.

### 2.4. Ningbo Independent External Validation Cohort

For independent external validation, we used the Ningbo First Hospital partition of the PhysioNet/Computing in Cardiology 2021 training data, which contains 34,905 ECGs [21]. These are 10 s, 12-lead recordings acquired at 500 Hz with a GE MUSE system; professional experts assigned the diagnoses [35]. The Ningbo partition should not be conflated with the earlier 10,646-record Chapman/Shaoxing cohort described by Zheng et al. [34]. For compatibility with the PTB-XL task, we resampled each recording to 100 Hz, represented it by 1000 samples per lead, and then applied the same record-wise, lead-wise normalization.

We required each eligible record to have a readable 12-lead waveform of the expected duration and at least one diagnostic code that could be mapped a priori to NORM, MI, STTC, CD, or HYP. The SNOMED-CT mapping contained one NORM code, five MI codes, five STTC codes, 11 CD codes, and three HYP codes; we provide all mappings in the source-data manifest. We excluded records carrying only diagnoses outside these target superclasses rather than forcing them into a target label. This procedure retained 15,931 ECGs, all of which we read successfully. The final counts were NORM = 6299, MI = 880, STTC = 6800, CD = 4688, and HYP = 637. Label counts overlap because one ECG can map to multiple superclasses.

We used Ningbo strictly once as an external test set. Before Ningbo inference, we locked the PTB-XL-trained checkpoint, preprocessing rule, and PTB-XL validation-derived thresholds. We did not use any Ningbo record or aggregate metric for architecture choice, hyperparameter tuning, recalibration, threshold selection, or candidate-model ranking. This design preserves Ningbo as an independent estimate of transfer under differences in acquisition, prevalence, patient composition, and diagnostic coding. Because the task is multi-label, class counts are not mutually exclusive. Table 2 summarizes the cohorts and class-wise label counts.

### 2.5. Patient-Level Demographic and Anthropometric Characteristics

We summarized the available patient-level metadata to describe the clinical composition of the PTB-XL and Ningbo cohorts. We used these variables only for descriptive cohort characterization and did not provide them as model inputs. We trained and evaluated the neural network using only the 12-lead ECG waveform and the five diagnostic superclass labels.

For PTB-XL, we treated age values above 120 years as missing because very old ages are privacy-coded in the public metadata. We summarized height and weight only after excluding physiologically implausible values and calculated BMI only when both valid height and valid weight were available. For Ningbo, age and sex were available, whereas height, weight, and BMI were not available in the extracted mapped metadata. Continuous variables are reported as mean ± standard deviation and median (interquartile range); height, weight, and BMI are available-case summaries and were not model inputs. Table 3 reports the cohort characteristics.

### 2.6. Representative ECG Examples and Label Distribution

**Figure 2:**
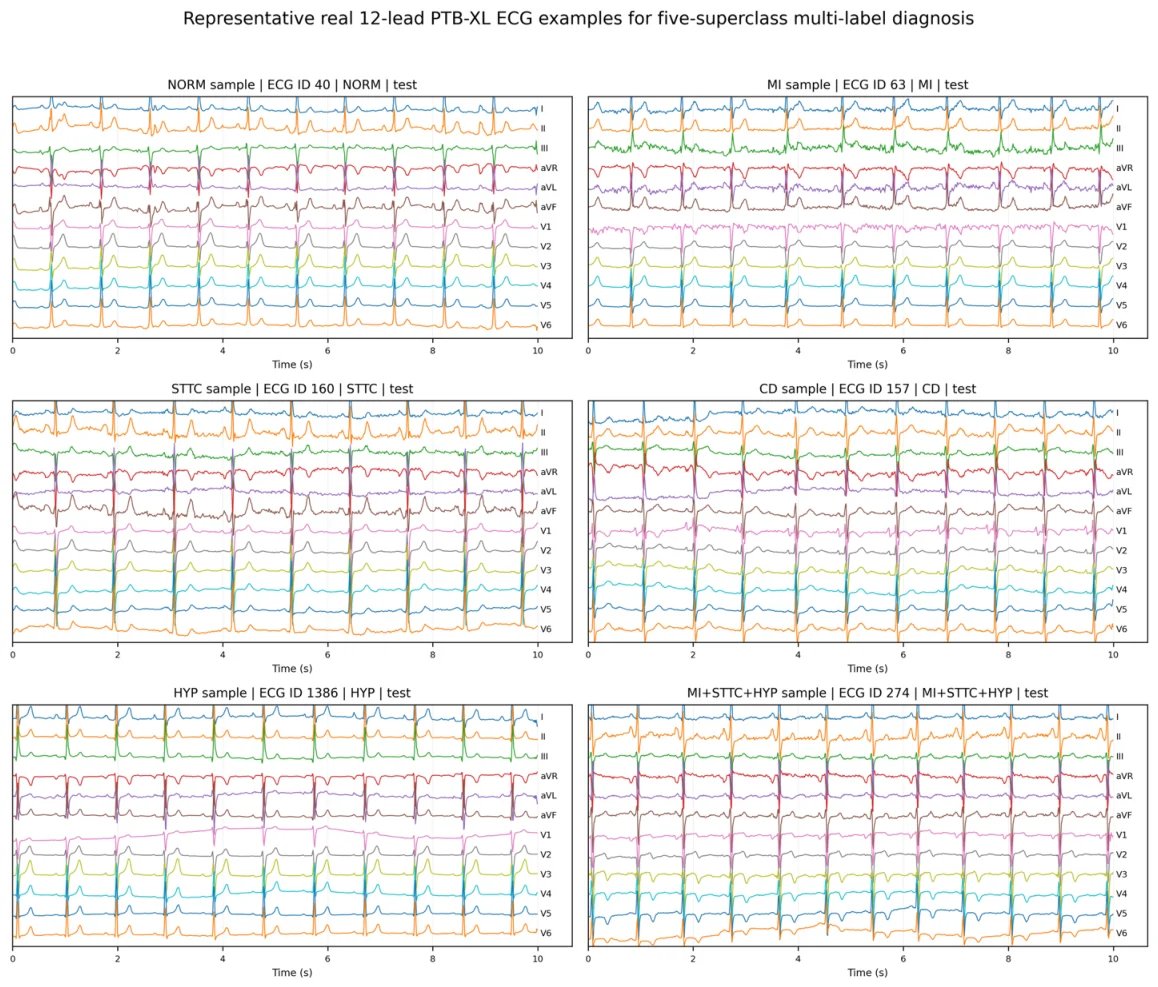
Representative multi-label PTB-XL ECGs.

Representative ECG examples were included to make the multi-label diagnostic setting visually explicit. Figure 2 presents real PTB-XL 12-lead ECG examples for the five diagnostic superclasses and one multi-label case. These examples illustrate that diagnostic evidence may appear in selected leads, occupy short temporal regions, and overlap across co-existing abnormalities.

**Figure 3:**
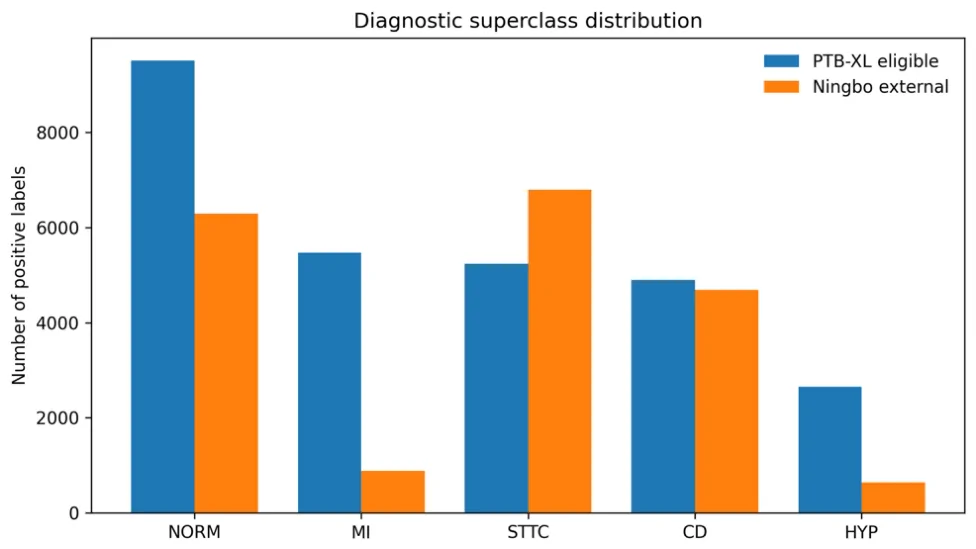
Diagnostic superclass distributions.

Figure 3 shows the diagnostic superclass distribution in PTB-XL and Ningbo. The different class profiles demonstrate the two main data challenges in this study: label imbalance and external source shift. These differences are important because the Ningbo cohort was not used for training or threshold selection.

## 3. Proposed Methodology

### 3.1. Problem Formulation

Let $X_{i}\in R^{1000\times 12}$ denote a 10 s 12-lead ECG record represented by 1000 temporal samples and 12 clinical leads. Let $y_{i}\in {\left\{0,1\right\}}^{5}$ be the corresponding diagnostic superclass vector for NORM, MI, STTC, CD, and HYP. Since these diagnostic categories may co-exist in the same recording, the task is formulated as multi-label classification rather than mutually exclusive multi-class classification. The objective is to learn a mapping from the ECG waveform to five independent diagnostic probabilities:

(1) $$f_{\theta }:R^{1000\times 12}\to {\left[0,1\right]}^{5},$$

where each output is produced by a sigmoid unit. Unlike a softmax classifier, the outputs are not constrained to sum to one, which allows the model to assign multiple diagnostic superclasses to the same ECG when appropriate.

### 3.2. Preprocessing and Signal Representation

We represented each ECG as a fixed 10 s 12-lead signal with 1000 temporal samples, giving an input tensor of size 1000×12. We retained the standard clinical lead order: I, II, III, aVR, aVL, aVF, and V1–V6. We used PTB-XL records in their 100 Hz representation and converted Ningbo records to the same fixed-length representation before inference. During the eligibility step summarized in Figure 1, we excluded records without a usable waveform or a valid mapping to at least one of the five target superclasses.

We deliberately kept preprocessing simple and identical for internal and external records. We did not use beat segmentation, handcrafted fiducial extraction, or external-cohort calibration statistics. We normalized each lead independently within the same ECG record:

(2) $${\tilde{x}}_{l,t}=\frac{x_{l,t}-\mu_{l}}{\sigma_{l}+10^{-8}},$$

where $x_{l,t}$ denotes the signal value for lead *l* at time index *t*, and $\mu_{l}$ and $\sigma_{l}$ are the corresponding mean and standard deviation of that lead within the same record. We replaced non-finite values with zero after normalization. Because the normalization is record-wise, our preprocessing does not require population means or standard deviations estimated from the validation, test, or external Ningbo cohort. This helps preserve the independence of the reported internal and external evaluations. We did not provide patient-level demographic or anthropometric variables, including age, sex, height, weight, and BMI, to the neural network; we used them only to describe the clinical composition of the cohorts. Table 4 summarizes the preprocessing and signal-representation protocol used for both PTB-XL and Ningbo evaluation.

### 3.3. Stacked InceptionTime–CNN–BiGRU–Transformer Architecture

**Figure 4:**
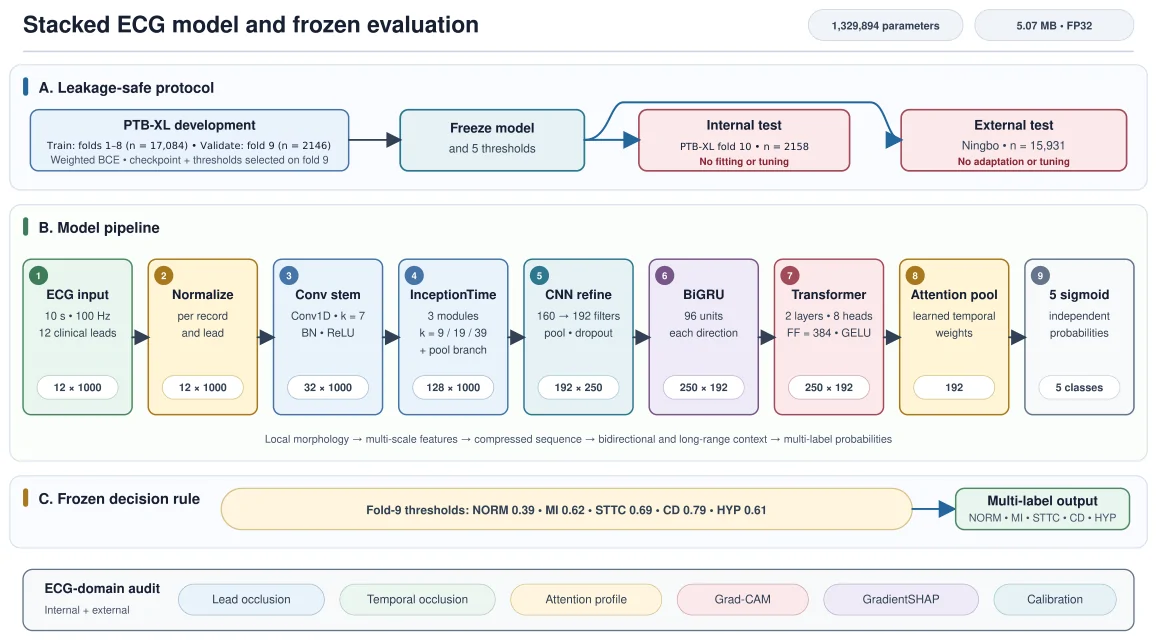
Model and evaluation workflow.

Figure 4 presents the complete workflow of our framework. The pipeline includes 12-lead ECG preprocessing, stacked InceptionTime–CNN–BiGRU–Transformer feature learning, validation-derived class-specific thresholding, official-fold PTB-XL model development, frozen Ningbo external validation, and ECG-domain interpretability. We designed the architecture to reflect the structure of ECG signals, where diagnostic information may be local, multi-scale, lead dependent, temporally distributed, or shared across co-existing abnormalities. Table 5 summarizes the layer-wise configuration of the stacked InceptionTime–CNN–BiGRU–Transformer architecture.

The first stage extracts local waveform features from the normalized ECG. The InceptionTime-style block then applies parallel temporal kernels with different receptive fields so that short-duration and longer-duration ECG patterns can be learned simultaneously. If *X* denotes the input representation, the multi-scale features can be expressed as

(3) $$Z_{\mathrm{inc}}=\left[{\mathrm{Conv}}_{9}\left(X\right);{\mathrm{Conv}}_{19}\left(X\right);{\mathrm{Conv}}_{39}\left(X\right);\mathrm{Pool}\left(X\right)\right],$$

where the concatenation combines features from multiple temporal scales. A hierarchical CNN module further refines the representation and compresses the temporal dimension:

(4) $$Z_{\mathrm{cnn}}=g_{\mathrm{cnn}}\left(Z_{\mathrm{inc}}\right).$$

The refined sequence is passed to a bidirectional GRU layer:

(5) $$H_{\mathrm{gru}}=\mathrm{BiGRU}\left(Z_{\mathrm{cnn}}\right),$$

which captures forward and backward temporal dependencies within the 10 s segment. The resulting representation is then processed by a Transformer encoder:

(6) $$H_{\mathrm{tr}}=\mathrm{TransformerEncoder}\left(H_{\mathrm{gru}}\right).$$

This stage models longer-range temporal interactions after convolutional downsampling. Temporal attention pooling is used to produce a weighted summary of the encoded sequence:

(7) $$\alpha_{t}=\frac{\exp(v^{⊤}\tanh\left(Wh_{t}\right))}{\sum_{s}\exp\left(v^{⊤}\tanh\left(Wh_{s}\right)\right)},\;c=\sum_{t}\alpha_{t}h_{t}.$$

Finally, a fully connected layer with sigmoid activation generates the five-superclass probability vector:

(8) $$\hat{y}=\sigma \left(W_{o}c+b_{o}\right).$$

The final stacked model contained 1,329,894 trainable parameters, corresponding to 5.07 MB in FP32 precision.

### 3.4. Computational Complexity and Measured Inference Cost

For an input sequence of length *T*, a one-dimensional convolution has leading cost $O(TkC_{\mathrm{in}}C_{\mathrm{out}})$, the BiGRU has cost O(Th(d+h)), and a Transformer layer has cost $O(T^{2}d+Td^{2})$. In this model, two max-pooling operations reduce the sequence from T=1000 to T=250 before the BiGRU and two-layer Transformer (d=192), limiting the quadratic attention term. Measured inference was performed on one NVIDIA A100-SXM4-40GB after warm-up and excluded file input/output and preprocessing. Table 6 reports empirical latency; these hardware-specific values should not be interpreted as bedside latency.

### 3.5. Loss Function and Threshold Optimization

Because each ECG can carry more than one diagnostic label, we trained the model using positive-class-weighted binary cross-entropy across the five diagnostic superclasses:

(9) $$L=-\frac{1}{N}\sum_{i=1}^{N}\sum_{k=1}^{5}\left[w_{k}^{+}y_{ik}\log\left({\hat{y}}_{ik}\right)+\left(1-y_{ik}\right)\log\left(1-{\hat{y}}_{ik}\right)\right],$$

where we calculated $w_{k}^{+}=N_{k}^{-}/N_{k}^{+}$ from folds 1–8 only. The resulting weights for NORM, MI, STTC, CD, and HYP were 1.249, 2.901, 3.081, 3.373, and 7.062, respectively. Thus, the rarer positive labels contributed more strongly to the training loss without changing their evaluation prevalence.

For each class *k*, we selected the decision threshold on fold 9 by exhaustive grid search:

(10) $$\tau_{k}^{*}=\underset{\tau \in \{0.10,0.11,\ldots ,0.90\}}{\arg\max}\;F1_{k}\left(\tau \right).$$

We resolved ties deterministically by retaining the first (lowest) threshold encountered by the ascending search. We fixed this rule and the grid in the analysis code and did not inspect fold 10 or Ningbo during threshold optimization.

Table 7 reports the class-specific fold-9 thresholds, which were frozen before PTB-XL fold-10 testing and Ningbo evaluation.

The five sigmoid outputs had different prevalence rates and score distributions; therefore, we preferred class-specific operating points to a uniform 0.5 threshold. In the frozen rule, for example, MI requires $p_{MI}\geq 0.62$, whereas CD requires $p_{CD}\geq 0.79$. We applied the resulting thresholds unchanged to all reported internal and external analyses. The validation-fold $F_{1}$-maximizing rule was used as a retrospective research choice for reproducible evaluation; it was not a clinically selected or validated operating point. Any prospective clinical evaluation would require prespecified operating points informed by local prevalence, workflow, safety priorities, and the relative consequences of false-positive and false-negative decisions.

### 3.6. Multi-Label Decision Algorithm

The proposed classifier is trained as a multi-label model rather than a mutually exclusive multi-class classifier. Algorithm 1 summarizes the decision strategy in compact mathematical form. The trained stacked InceptionTime–CNN–BiGRU–Transformer model first produces five sigmoid probabilities, and the validation-derived class-specific thresholds in Table 7 are then applied to obtain the final label vector.
**Algorithm 1** Multi-label ECG classification using validation-tuned sigmoid decisions**Input:** ECG record $X_{i}\in R^{1000\times 12}$, trained model $f_{\theta^{*}}$, thresholds $\tau =(\tau_{1},\ldots ,\tau_{5})$, class set C={NORM,MI,STTC,CD,HYP}**Output:** Multi-label prediction vector ${\hat{y}}_{i}\in {\left\{0,1\right\}}^{5}$1:**for** ℓ=1,…,12 **do**2:${\tilde{x}}_{i,t,\ell }\leftarrow \frac{x_{i,t,\ell }-\mu_{i,\ell }}{\sigma_{i,\ell }+10^{-8}}$ for all time indices *t***end for**3:${\hat{p}}_{i}\leftarrow f_{\theta^{*}}\left({\tilde{X}}_{i}\right)=\sigma \left(W_{o}c_{i}+b_{o}\right)$4:**for** k=1,…,5 **do**5:${\hat{y}}_{ik}\leftarrow I\left({\hat{p}}_{ik}\geq \tau_{k}\right)$**end for**6:**return** ${\hat{y}}_{i}=\left[{\hat{y}}_{i1},{\hat{y}}_{i2},{\hat{y}}_{i3},{\hat{y}}_{i4},{\hat{y}}_{i5}\right]$

### 3.7. Interpretability Design

We designed the interpretability strategy to remain close to the structure of clinical ECG review. In the lead-occlusion analysis, we masked one lead at a time and measured the corresponding change in class probability. In the temporal-occlusion analysis, we masked short time windows and quantified the resulting probability drop. We used attention profiling to summarize the temporal weights learned by the model and identify which regions of the encoded sequence contributed most to the final prediction.

In addition, we computed Grad-CAM temporal localization using the last Inception block as the target convolutional representation. We used gradients of the class-specific logit to weight the temporal activation maps and upsampled the resulting one-dimensional heatmap to the original ECG length. We interpret Grad-CAM as temporal localization support rather than proof of causal electrophysiology and assess lead-specific evidence separately through lead occlusion. These analyses provide distinct audit views at their respective analytical levels, and quantitative concordance between them was not evaluated.

## 4. Experimental Setup

### 4.1. Training Protocol

Our experimental protocol followed the official PTB-XL partitioning described in Section 2. We selected the best checkpoint using the validation fold only. For model optimization, we used adaptive gradient-based training with validation monitoring, following the standard Adam/AdamW family of optimization strategies for deep neural networks [41,42]. After model selection, we froze both the checkpoint and the validation-derived thresholds before inference on the PTB-XL test fold and before any evaluation on the Ningbo external cohort.

**Figure 5:**
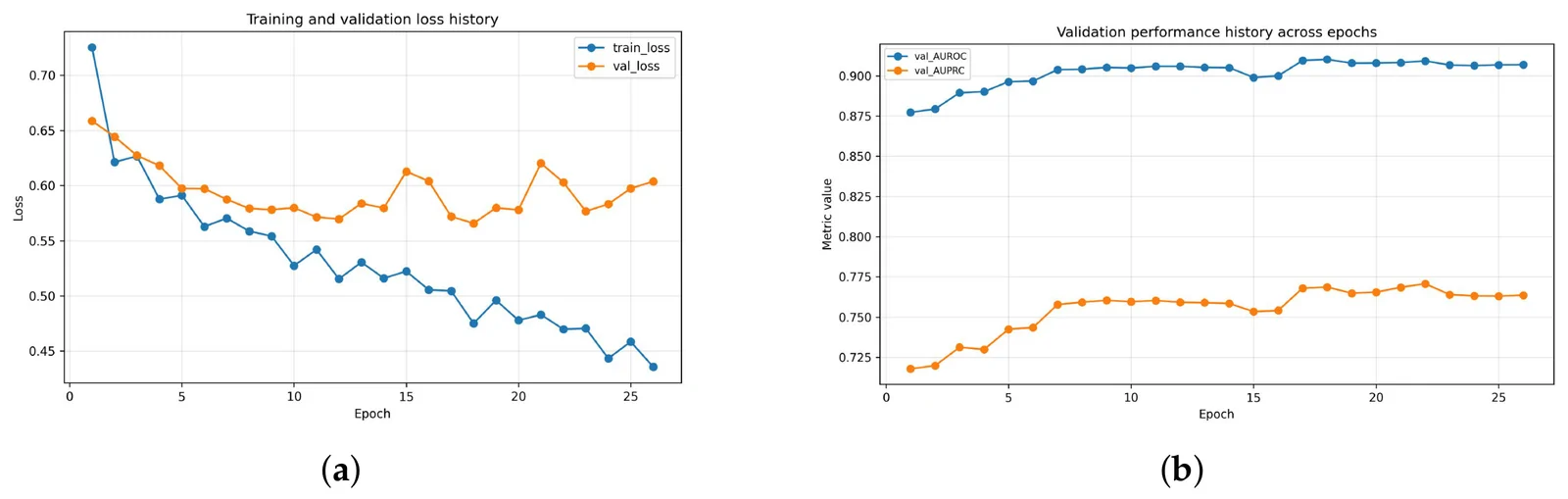
Training dynamics. (**a**) Training and validation loss. (**b**) Validation AUROC and AUPRC.

We adopted this design to reduce the risk of optimistic evaluation. Neither the PTB-XL test fold nor the Ningbo cohort contributed to parameter learning, hyperparameter selection, calibration, threshold optimization, or post hoc decision adjustment. Thus, our internal test results reflect performance on an unseen PTB-XL fold, whereas the Ningbo results reflect performance under an independent source shift with the same frozen model and thresholds. Figure 5 shows the training and validation dynamics.

### 4.2. Post Hoc Architectural Analyses

We conducted two complementary post hoc analyses: a protocol-matched exact-parent ablation to isolate component contributions within the primary architecture and an architecture-sensitivity analysis to evaluate performance across independently parameterized alternatives. Both architectural analyses were designated post hoc because they were not part of the prespecified primary evaluation and did not alter the frozen primary checkpoint. Neither analysis used Ningbo outcomes for model training, ranking, or selection.

#### 4.2.1. Exact-Parent Ablation

We evaluated five protocol-matched PTB-XL models: CNN-only, InceptionTime-CNN, InceptionTime-CNN-BiGRU, InceptionTime–CNN–Transformer, and the complete InceptionTime–CNN–BiGRU–Transformer architecture. The exact-parent ablation comprised the full architecture and three nested variants obtained by removing the Transformer, the BiGRU, or both sequence modules while preserving the parent interfaces. CNN-only was included as an additional protocol-matched comparator. All five models used identical records, official folds, preprocessing, augmentation, loss, seed, batch size 16, recovered optimization schedule, checkpoint-selection rule, and validation-derived thresholds. Each comparator was evaluated against the matched full architecture using 2000 paired record-level bootstrap resamples, with Holm adjustment across six endpoints within each comparison.

#### 4.2.2. Architecture Sensitivity Analysis

The complementary architecture sensitivity analysis evaluated seven separately parameterized configurations: CNN-only, CNN–BiGRU, CNN–Transformer, InceptionTime–CNN, a 1,270,822-parameter full-stack sensitivity implementation, InceptionTime–CNN–Transformer without BiGRU, and InceptionTime–CNN with attention pooling. These configurations followed a common PTB-XL data and evaluation protocol but were not nested removals from the 1,329,894-parameter frozen primary model. Accordingly, the exact-parent ablation assesses component effects within the locked parent architecture, whereas the sensitivity analysis assesses broader dependence on architectural choices and cannot isolate the incremental effect of an individual module. This distinction supports transparent robustness reporting and avoids interpreting separately parameterized comparisons as component-level evidence [4,5].

### 4.3. Evaluation Metrics

We evaluated performance using complementary ranking, threshold-dependent, and multilabel metrics because each metric answers a different diagnostic question. AUROC and AUPRC evaluate the quality of the predicted probabilities before thresholding. We interpreted AUPRC alongside AUROC because precision–recall analysis is particularly informative when positive labels are imbalanced [43]. After applying the validation-derived class-specific thresholds, we summarized decision-level performance using precision, sensitivity/recall, specificity, and F1-score. For each superclass, we computed these quantities from the one-vs-rest counts of true positives (TP), false positives (FP), false negatives (FN), and true negatives (TN):

(11) $$\mathrm{Precision}=\frac{\mathrm{TP}}{\mathrm{TP}+\mathrm{FP}},$$

(12) $$\mathrm{Sensitivity}=\mathrm{Recall}=\frac{\mathrm{TP}}{\mathrm{TP}+\mathrm{FN}},$$

(13) $$\mathrm{Specificity}=\frac{\mathrm{TN}}{\mathrm{TN}+\mathrm{FP}},$$

(14) $$F1=\frac{2\times \mathrm{Precision}\times \mathrm{Recall}}{\mathrm{Precision}+\mathrm{Recall}}.$$

We used exact match and Hamming loss to evaluate complete multilabel decision quality. Exact match requires the full five-label prediction vector to match the reference vector for a record, whereas Hamming loss measures the fraction of incorrect record–class decisions:

(15) $$\mathrm{Hamming}\;\mathrm{loss}=\frac{1}{NK}\sum_{i=1}^{N}\sum_{k=1}^{K}I\left({\hat{y}}_{ik}\neq y_{ik}\right),$$

where *N* is the number of ECG records and K=5 is the number of diagnostic superclasses. For readability, we derived label-wise accuracy as 100×(1−Hammingloss) and report it only as the percentage of individual superclass decisions that were correct; it does not replace F1-score, sensitivity, specificity, calibration, or class-wise error analysis. Table 8 summarizes the clinical interpretation of the multi-label evaluation metrics.

We report both macro- and micro-averaged metrics. Macro-averaging gives equal weight to each diagnostic superclass and is therefore useful for identifying behavior on minority classes. Micro-averaging pools all label decisions and is more strongly influenced by frequent classes. Reporting both AUROC/AUPRC and F1-based metrics is important in external validation because a model may retain useful ranking ability while fixed-threshold decisions degrade under source shift. This combined metric strategy provides a more complete assessment of internal discrimination, threshold transfer, and multi-label decision reliability.

We assessed calibration independently for each class. Expected calibration error (ECE) used 10 fixed equal-width probability bins over [0,1] and weighted the absolute difference between mean predicted probability and observed prevalence by bin occupancy. Brier score was the mean squared probability error. We calculated macro-ECE and macro-Brier as unweighted means across the five classes and did not fit a recalibration model.

We estimated uncertainty using a nonparametric ECG-level bootstrap with 2000 resamples, sampling the same number of ECG records with replacement within each cohort. We kept the complete five-label vector and its five predicted probabilities together in each resampled record. Using seed 20260722, we computed percentile 95% confidence intervals for macro-AUROC, macro-AUPRC, micro-F1, macro-F1, exact match, Hamming loss, macro-ECE, and macro-Brier. Paired fold-10 bootstraps compared each exact-parent variant with the matched full architecture and compared the frozen primary checkpoint with the sensitivity-analysis CNN-only model; Holm adjustment was applied across six endpoints within each comparison. We did not perform cross-paper significance tests because predictions and record identities from published comparators were unavailable and their tasks were not matched.

## 5. Results

### 5.1. Internal PTB-XL Test Performance

We first evaluated the frozen primary model on the official PTB-XL fold-10 test set. It achieved macro-AUROC 0.9083 (95% CI 0.8998–0.9164) and macro-AUPRC 0.7741 (0.7565–0.7912). At the fold-9-derived thresholds, micro-F1 was 0.7517 (0.7392–0.7640), macro-F1 0.7130 (0.6987–0.7272), weighted-F1 0.7577, exact match 0.5524 (0.5315–0.5737), and Hamming loss 0.1390 (0.1316–0.1464), corresponding to a label-wise accuracy of 86.10%.

Table 9 reports the class-wise internal test results. The model achieved strong AUROC values for NORM, MI, STTC, and CD, while HYP remained the most difficult class, particularly in terms of AUPRC and F1-score. This behavior is expected in a multi-label ECG setting because HYP has fewer positive examples and can overlap with other morphological or repolarization patterns. The class-wise results therefore support the need to report both ranking metrics and threshold-dependent metrics rather than relying on a single aggregate score.

**Figure 6:**
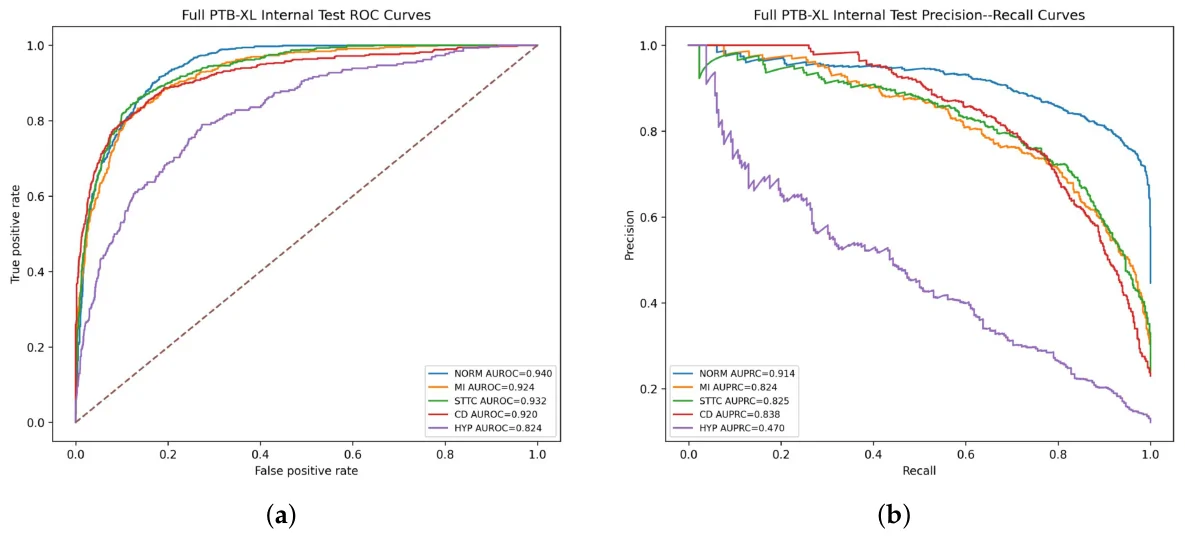
Internal ranking curves. (**a**) ROC curves. (**b**) Precision–recall curves.

Figure 6 shows the ROC and precision–recall curves for the PTB-XL test fold. The ROC curves demonstrate consistent discrimination across the five superclasses, whereas the precision–recall curves provide a more prevalence-sensitive view of the same predictions. The lower precision–recall behavior for HYP is consistent with the class-wise metrics in Table 9.

**Figure 7:**
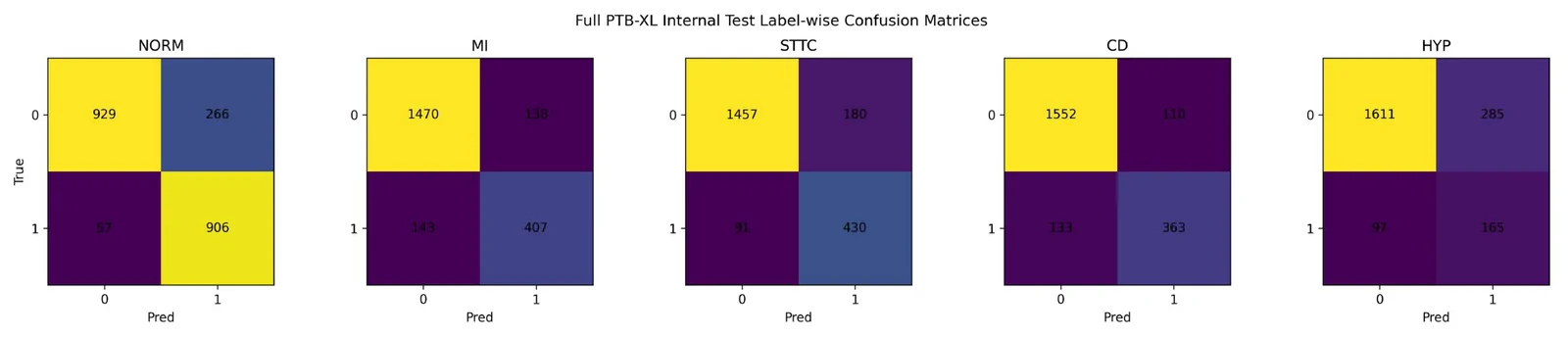
Internal one-vs-rest confusion matrices.

Figure 7 illustrates the one-vs-rest confusion matrices on the PTB-XL internal test fold. These matrices summarize thresholded judgments created by the multi-label rule tuned on the validation set in Algorithm 1. They provide more direct insight into false-positive and false-negative behavior than ranking measures alone.

Table 10 converts the internal one-vs-rest confusion matrices into a class-wise clinical error profile. NORM had high sensitivity, which meant that most truly normal records were accurately detected, but its lower specificity indicated that some abnormal records were labeled as normal by the multi-label decision process. HYP showed the weakest F1 and AUPRC, consistent with lower prevalence and overlap with repolarization or voltage-related patterns. This is precisely why the paper reports class-wise results rather than a single F1 value.

### 5.2. Post Hoc Exact-Parent Ablation

Table 11 reports the five protocol-matched models used to test the exact primary architecture.

BiGRU removal reduced micro-F1 by 0.0140 (95% CI −0.0226 to −0.0044; Holm p=0.040) and exact match by 0.0218 (95% CI −0.0375 to −0.0055; p=0.040), while increasing Hamming loss by 0.0088 (95% CI 0.0031–0.0138; p=0.036). Transformer removal, removal of both sequence modules, and CNN-only produced no Holm-significant differences from the matched full architecture.

### 5.3. Complementary Post Hoc Architecture Sensitivity Analysis

This complementary analysis evaluated seven separately parameterized configurations under common PTB-XL data and evaluation conditions. It was distinct from the exact-parent ablation: the 1,270,822-parameter full-stack sensitivity implementation was not the 1,329,894-parameter frozen primary checkpoint, and none of the sensitivity configurations was used for Ningbo evaluation or retrospective model selection.

Table 12 summarizes the separately trained sensitivity configurations. CNN-only achieved the highest macro-AUROC and macro-F1, whereas the post hoc full-stack sensitivity implementation achieved the highest macro-AUPRC, micro-F1, and exact match within this post hoc set. These results describe broader architecture sensitivity and do not isolate the incremental contribution of the recurrent or Transformer modules within the frozen primary architecture.

**Figure 8:**
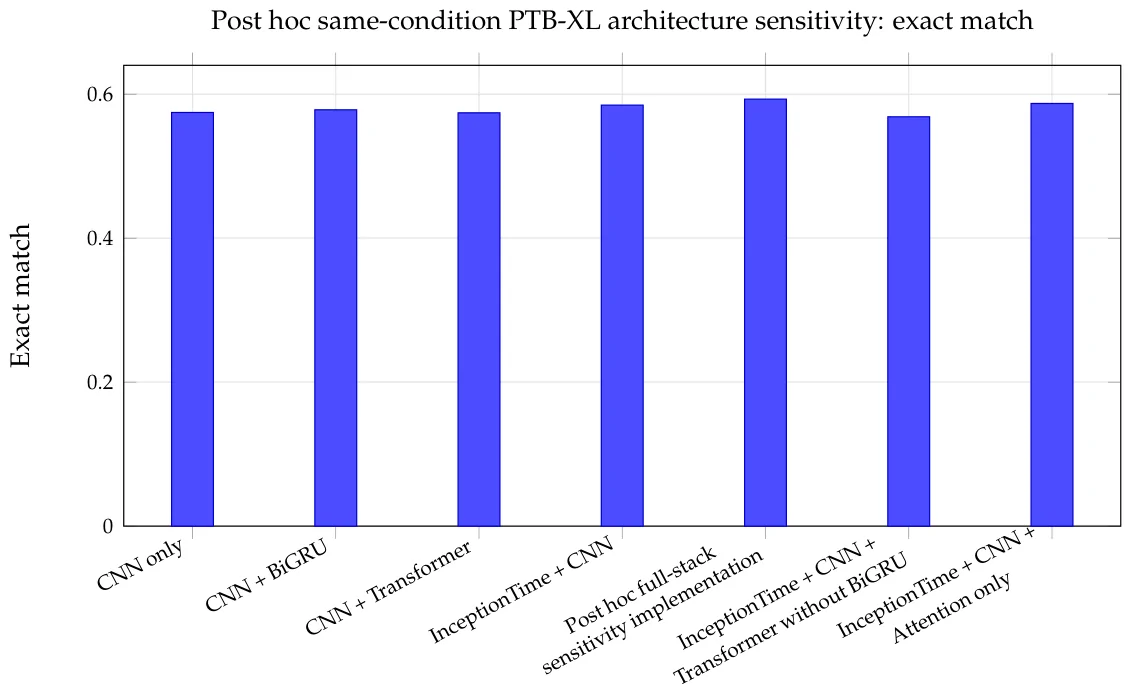
Exact match across architecture sensitivity configurations.

The 1,270,822-parameter post hoc full-stack sensitivity implementation is distinct from the 1,329,894-parameter frozen primary checkpoint. Figure 8 compares exact match across the seven sensitivity configurations.

Because complete aligned predictions were preserved for the frozen primary and sensitivity-analysis CNN-only runs, we performed a paired ECG-level bootstrap comparison on PTB-XL fold 10. Differences are calculated as primary minus CNN-only, with Holm adjustment across the six metrics. Table 13 reports the results. CNN-only performed significantly better on five of six Holm-adjusted endpoints; macro-AUPRC did not differ significantly. This finding does not provide a basis for retrospective replacement of the prespecified model, whose checkpoint and external evaluation remained unchanged.

### 5.4. Independent Ningbo External Validation

We performed the Ningbo experiment only after freezing the PTB-XL checkpoint and all class-specific thresholds. Algorithm 2 summarizes the external-validation protocol; we place it here to keep the testing procedure close to the corresponding results. This protocol distinguishes external testing from model development: we did not use Ningbo records for retraining, recalibration, threshold optimization, or post hoc decision adjustment.

Under this frozen protocol, we obtained a macro-AUROC of 0.8885 (95% CI 0.8847–0.8923) and a macro-AUPRC of 0.6487 (0.6383–0.6601) on 15,931 Ningbo ECGs. At the unchanged PTB-XL thresholds, micro-F1 was 0.6507 (0.6458–0.6556), macro-F1 0.5592 (0.5538–0.5648), weighted-F1 0.7169, exact match 0.4090 (0.4015–0.4164), and Hamming loss 0.1968 (0.1938–0.1999), corresponding to label-wise accuracy of 80.32%. The separation between ranking and fixed-threshold metrics indicates retained diagnostic signal with imperfect operating-point and probability transfer. It does not establish clinical readiness.

Table 14 compares overall internal and external multi-label performance.

Table 15 reports class-wise Ningbo performance at the frozen PTB-XL validation thresholds.

Using 2000 ECG-level bootstrap resamples, Table 16 reports 95% confidence intervals for the frozen primary model in both cohorts.
**Algorithm 2** Frozen Ningbo external validation and ECG-domain audit**Input:** Ningbo records $D_{\mathrm{ext}}$, frozen model $f_{\theta^{*}}$, frozen thresholds τ, class set C**Output:** External performance metrics and representative interpretability outputs  1:Map Ningbo diagnostic statements conservatively to the five PTB-XL diagnostic superclasses in C.  2:Remove records without a valid mapped target superclass or without a usable waveform.  3:Keep $f_{\theta^{*}}$ and τ fixed; do not retrain, recalibrate, or retune thresholds on Ningbo.  4:**for all** $X_{j}\in D_{\mathrm{ext}}$ **do**  5:Normalize each lead using the same record-wise rule used for PTB-XL.  6:${\hat{p}}_{j}\leftarrow f_{\theta^{*}}\left({\tilde{X}}_{j}\right)$  7:**for** k=1,…,5 **do**  8:${\hat{y}}_{jk}\leftarrow I\left({\hat{p}}_{jk}\geq \tau_{k}\right)$**end for****end for**  9:Compute AUROC, AUPRC, precision, sensitivity, specificity, F1-score, exact match, and Hamming loss.10:For representative external cases, compute lead occlusion, temporal occlusion, attention profiles, and Grad-CAM temporal localization.11:**return** External metric tables, ROC/PR curves, class-wise analysis, and ECG-domain audit figures

**Figure 9:**
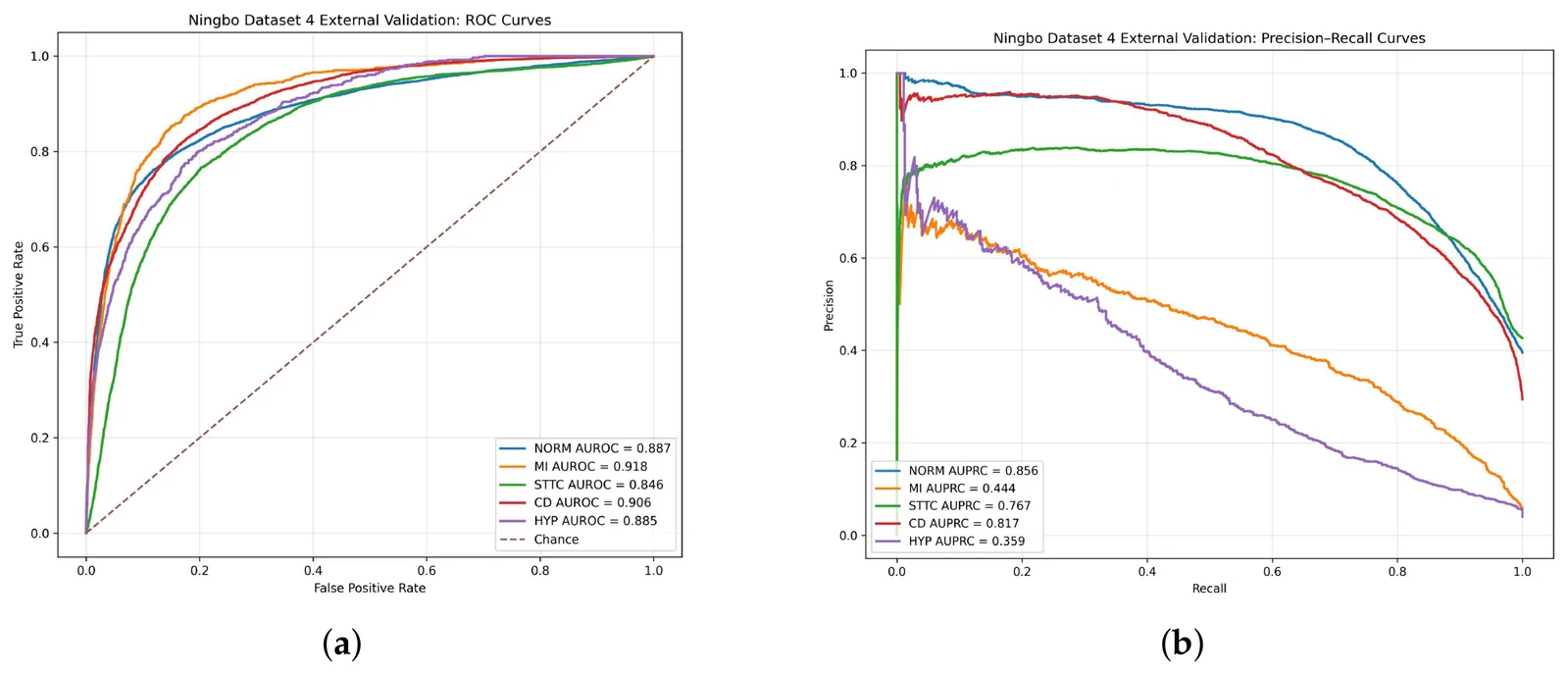
External ranking curves. (**a**) Ningbo ROC curves. (**b**) Ningbo precision–recall curves.

**Figure 10:**
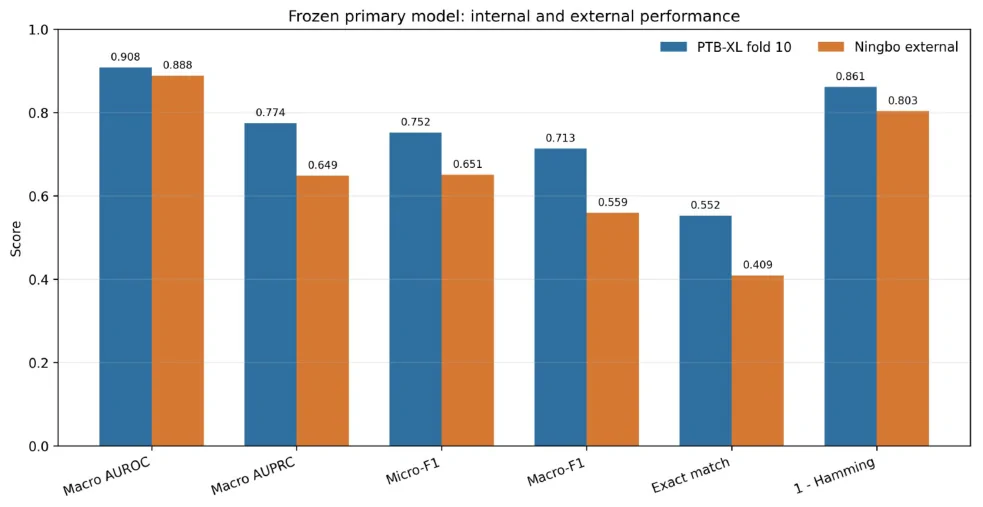
Internal–external performance comparison.

Figure 9 shows the external ROC and precision–recall curves. The ROC curves show that discrimination was maintained for all five superclasses; however, the precision-recall curves suggest a more pronounced effect of class imbalance on MI and HYP in the Ningbo cohort. Figure 10 also illustrates the internal and external performance profiles, highlighting the relevance of reporting both ranking and thresholded metrics in multi-label ECG classification.

### 5.5. Calibration Analysis

Expected calibration error (ECE) used 10 equal-width bins, with lower values indicating better calibration. Table 17 reports per-class calibration for both cohorts. We obtained an internal macro-ECE of 0.1241 (95% CI 0.1198–0.1297) and a macro-Brier score of 0.1206 (0.1156–0.1254). Under external transfer, macro-ECE increased to 0.2095 (0.2070–0.2121) and macro-Brier to 0.1735 (0.1715–0.1756). We observed the largest external calibration errors for MI and HYP, the two least prevalent Ningbo labels. Thus, retained external AUROC should not be interpreted as reliable absolute risk estimation.

**Figure 11:**
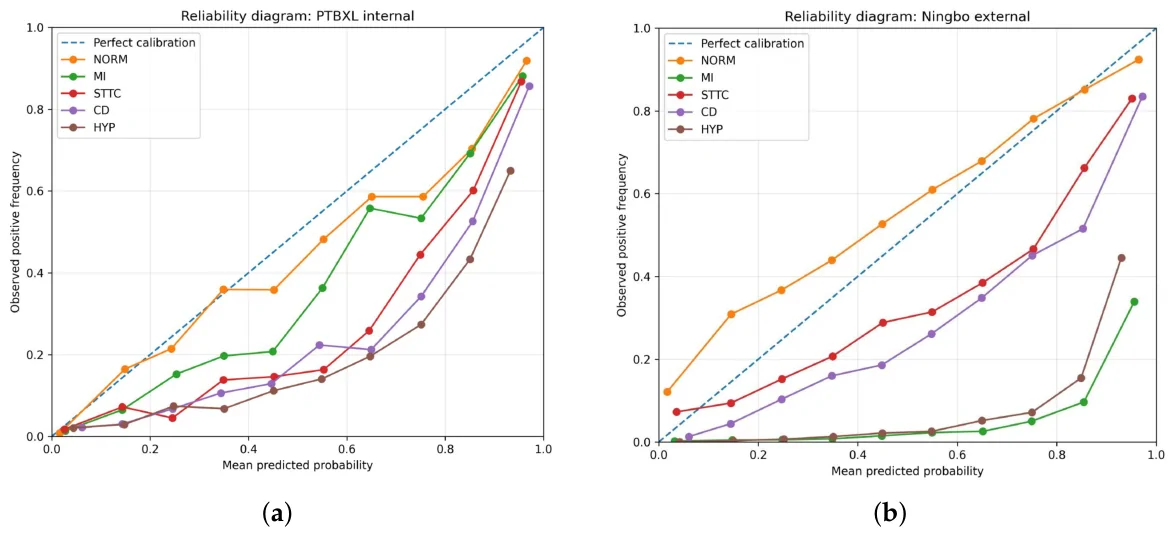
Reliability diagrams. (**a**) PTB-XL fold 10. (**b**) Ningbo external.

We also inspected calibration visually using the reliability diagrams in Figure 11. The diagrams compare mean predicted probability with observed positive frequency across 10 probability bins for each superclass. The PTB-XL plot provides the internal reference, whereas Ningbo shows the effect of source shift under the same frozen probabilities. We performed no external recalibration.

The calibration deterioration, together with low external MI precision (0.1801) and HYP precision (0.1396), is a central limitation. The frozen model may support retrospective ranking and audit experiments, but local recalibration, threshold review, and prospective clinical validation would be required before any decision-support use.

### 5.6. Demographic Subgroup Analysis

We treated the age- and sex-stratified analyses as descriptive; neither variable was a model input or tuning criterion. We computed the values listed in Table 18 directly from aligned per-record predictions. Female groups had higher micro-F1 and lower Hamming loss than male groups in both cohorts, while younger groups had higher exact match and lower Hamming loss than older groups. These differences may reflect prevalence, comorbidity, acquisition, or annotation differences, and we did not adjust them for confounding. We excluded 32 privacy-coded or missing PTB-XL ages, 37 missing Ningbo ages, and 13 unknown Ningbo sex values only from the corresponding stratified rows.

### 5.7. ECG-Domain Interpretability and Localization Evidence

**Figure 12:**
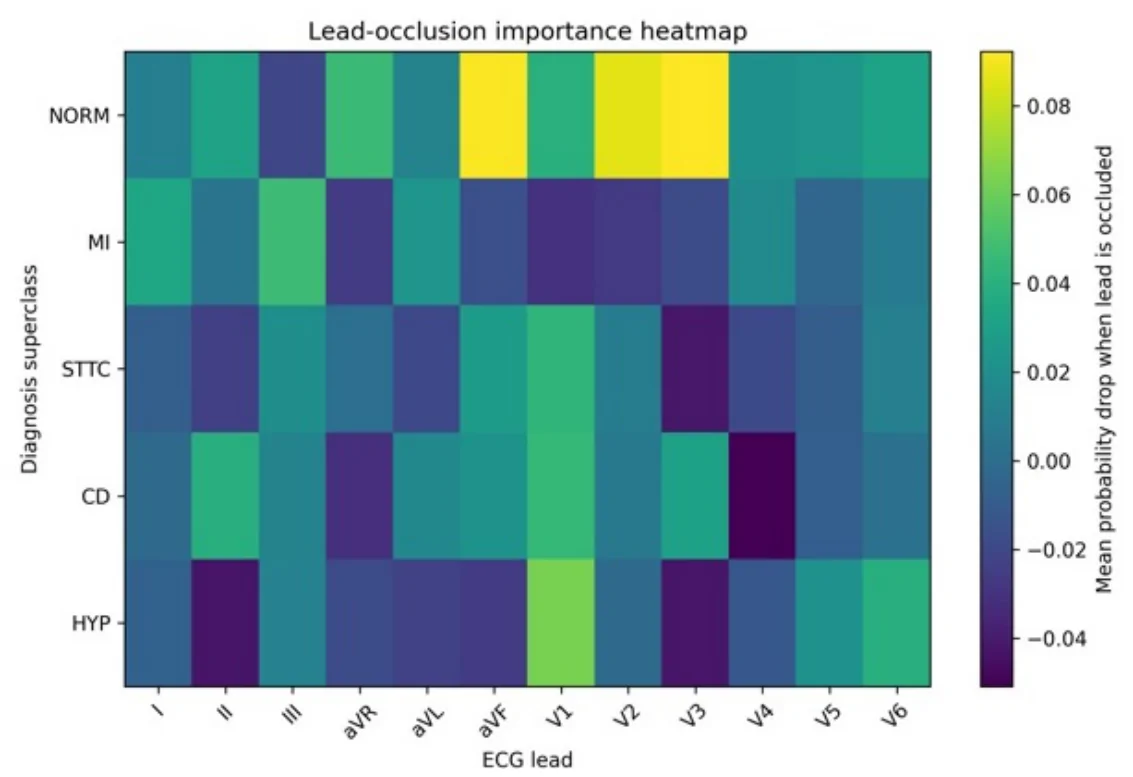
Lead-occlusion importance map.

**Figure 13:**
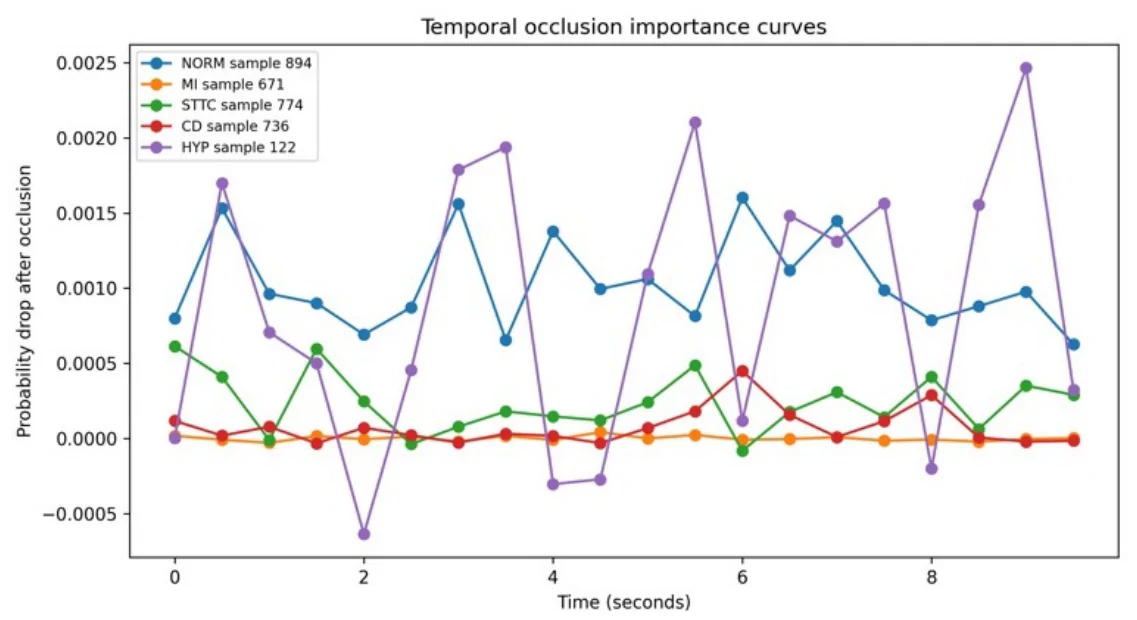
Temporal-occlusion curves.

**Figure 14:**
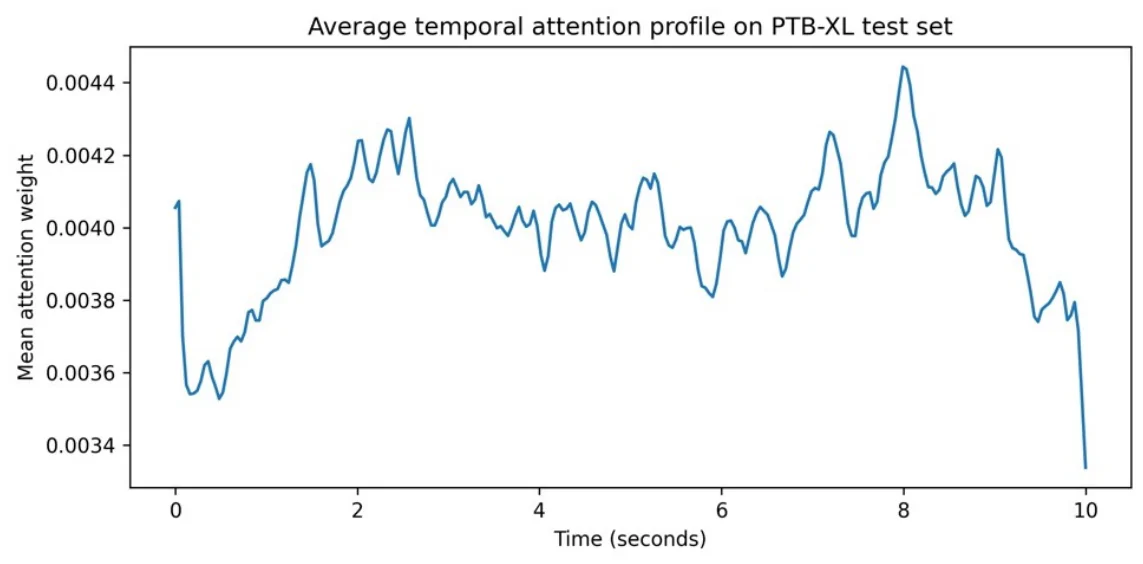
Temporal attention profile.

Figure 12, Figure 13 and Figure 14 summarize the ECG-domain localization analysis. The lead-occlusion map indicates which lead has the largest effect on class probabilities after masking. In the temporal occlusion curves, expected probabilities are plotted vs. the progressive occlusion of ECG windows. The attention profile shows the temporal weights used by the model on the 10 s recording. These perspectives are aligned with ECG reading practice, as they refer to leads and waveform intervals rather than abstract feature-importance ratings alone. The three components are plotted as distinct larger figures to preserve the axis names, legends, and color-bar information.

**Figure 15:**
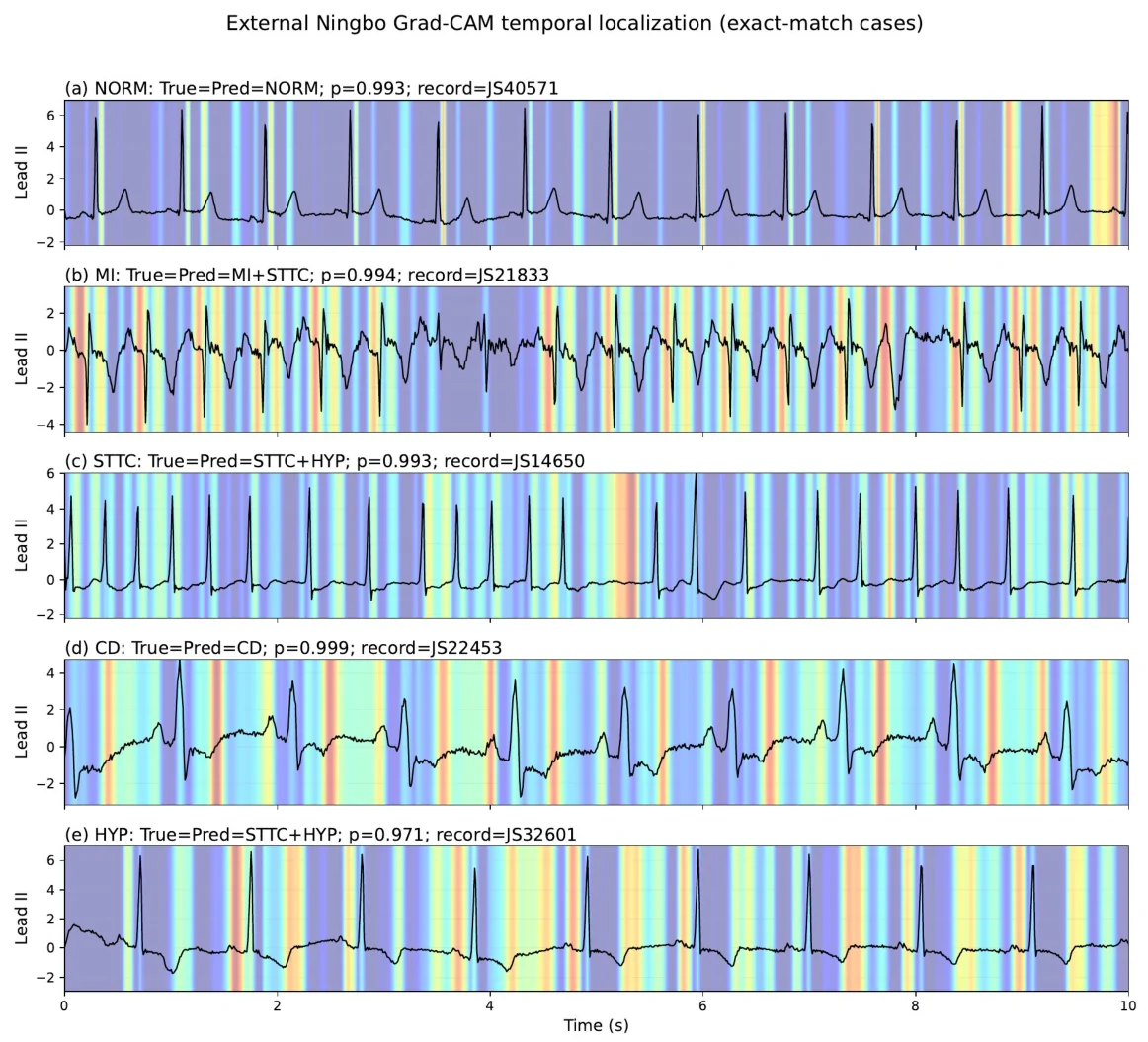
External Ningbo Grad-CAM examples.

We extended the localization analysis to the independent Ningbo cohort using the exact-match, high-confidence external instances shown in Figure 15. For each target superclass (NORM, MI, STTC, CD, and HYP), we selected one representative case. The selected target-class sigmoid probabilities ranged from 0.971 to 0.999; we show these probabilities in the figure titles as case-level model confidence values, not as dataset-level performance measures. Warmer regions indicate temporal intervals that contributed more strongly to the selected diagnostic superclass. In multi-label ECGs, the heatmap describes only the target class displayed; other co-existing labels would require separate class-specific Grad-CAM maps. Because the heatmap is one-dimensional, we interpret it as temporal localization rather than direct lead-specific attribution. We provide lead-specific evidence separately through occlusion and the supplementary GradientSHAP lead-attribution analysis in Appendix A.

The lead-occlusion analysis gave class-specific insight into the contribution of individual leads. We observed the largest probability decreases for NORM around aVF, V2, and V3. For MI, we found the largest effects in leads III, I, and aVL. The most influential leads for STTC were V1, aVF, and III, whereas the largest effects for CD occurred in V1, II, and V3. For HYP, we observed the largest effect in V1, followed by V6 and V5. These post hoc associations provide model-audit evidence only; they neither establish causal electrophysiology nor demonstrate clinical validity.

Lead occlusion and GradientSHAP address related but distinct model-audit questions at different analytical levels. Table 19 reports mean probability changes after lead masking across positive examples for each class, whereas GradientSHAP presents case-specific attributions for a small set of selected representative external ECGs. Differences in the leads emphasized by these analyses are therefore possible. Because cohort-level concordance was not quantitatively evaluated, the representative GradientSHAP findings should not be interpreted as validating the cohort-level lead-occlusion results; we present the two methods as complementary audit perspectives.

## 6. Discussion and Conclusions

### 6.1. Main Findings

We obtained macro-AUROC values of 0.9083 internally and 0.8885 externally, while external macro-AUPRC, micro-F1, and exact match decreased to 0.6487, 0.6507, and 0.4090. External macro-ECE increased to 0.2095, with particularly large errors for MI and HYP. The model therefore retained useful ranking information under source shift but did not transfer as a calibrated clinical probability estimator or a validated clinical operating rule. Our main contribution is a leakage-controlled evaluation workflow with traceable thresholds, uncertainty intervals, and a structured failure-mode audit. The stacked primary architecture was prespecified to integrate multiscale morphology, bidirectional temporal context, and global sequence interactions, and its frozen checkpoint was retained to avoid outcome-driven model replacement after test results became available. Its designation as the primary model therefore reflected the prespecified evaluation pathway rather than demonstrated superiority over simpler alternatives.

In the protocol-matched ablation, CNN-only yielded numerically better performance than the matched full architecture on five of six endpoints, but none of the paired differences was significant after Holm adjustment. Among the module-removal variants, Holm-significant deterioration in micro-F1, exact match, and Hamming loss occurred only when the BiGRU was removed while the Transformer was retained. Evidence of a component effect was therefore confined to this specific recurrent-module removal under the locked parent protocol. The complementary post hoc sensitivity analysis provided broader architectural context across seven separately parameterized configurations. CNN-only achieved the highest macro-AUROC (0.9142) and macro-F1 (0.7293), whereas the 1,270,822-parameter full-stack sensitivity implementation achieved the highest macro-AUPRC (0.7820), micro-F1 (0.7702), and exact match (0.5931). In the paired comparison with the frozen primary checkpoint, CNN-only outperformed on five of six Holm-adjusted endpoints; macro-AUPRC did not differ significantly. Because the sensitivity full stack differed from the 1,329,894-parameter primary architecture, these findings describe broader architecture sensitivity rather than exact-parent component effects and were not used for retrospective model replacement or external model selection.

### 6.2. Comparison with Previous Studies

NR denotes a result not reported in a directly comparable form. Table 20 places our study alongside recent multi-label ECG work. We use a protocol-aware comparison because accuracy, macro-F1, and AUROC are not interchangeable, and scores obtained from different label spaces, source mixtures, and evaluation protocols cannot be interpreted as paired head-to-head estimates. In particular, the 2026 CNN–BiLSTM–Transformer study reported a macro-F1 of 0.8301 and an accuracy of 0.9124 for a 27-label Challenge task [33]; these values are not directly comparable with our five-superclass PTB-XL fold-10 and frozen Ningbo evaluation.

### 6.3. Limitations

This study should be interpreted as a retrospective research and model-audit prototype rather than a clinically validated decision-support system. The official-fold development protocol, frozen external evaluation, and structured audit provide a reproducible assessment, but prospective cardiologist-in-the-loop evaluation has not been performed. External validation was limited to one public source, and harmonizing heterogeneous SNOMED-coded diagnoses into five broad superclasses may introduce label uncertainty. External calibration deteriorated under dataset shift, particularly for MI and HYP, and the validation-fold $F_{1}$-maximizing thresholds represent a retrospective research choice rather than clinically selected operating points. Before any clinical decision-support use, local recalibration, prespecified operating-point selection, safety and error-cost assessment, and prospective multicenter evaluation involving clinicians would be required. The post hoc architectural analyses used a single official PTB-XL split and a single random seed and were not externally evaluated; their findings are therefore condition-specific and should not be interpreted as universal architectural rankings.

Overall, the study provides a compact and interpretable ECG framework whose diagnostic ranking remained useful under external source shift despite deterioration in calibration. The frozen external evaluation, exact-parent ablation, and complementary sensitivity analysis strengthen the model audit without altering the prespecified primary model. Prospective multicenter validation, local recalibration, clinically informed operating-point selection, and cardiologist-in-the-loop safety assessment remain necessary before clinical use.

## Figures and Tables

**Table 1:** Clinical interpretation of PTB-XL diagnostic superclasses.

Class	Clinical Meaning	Common ECG Evidence Reviewed by Clinicians	Clinical Caveat
NORM	Recordings without the target diagnostic abnormalities represented by the five-superclass task.	Preserved overall rhythm and morphology without target infarction, ST/T, conduction, or hypertrophy patterns.	A NORM output should not be interpreted as absence of all possible cardiac disease; it is normal only relative to the target label set.
MI	Myocardial infarction-related ECG patterns represented in PTB-XL diagnostic statements.	Pathological Q-wave patterns, infarction-related morphology, and regional lead changes.	MI may co-occur with STTC or CD; external fixed-threshold performance should be interpreted with local prevalence and annotation practice.
STTC	ST-segment and T-wave change superclass.	ST-segment deviation, T-wave inversion, or abnormal repolarization morphology in anatomically related leads.	ST/T changes can overlap with ischemia, hypertrophy strain, medication or electrolyte effects, or secondary changes due to conduction disturbance.
CD	Conduction disturbance superclass.	Bundle-branch block patterns, widened QRS morphology, atrioventricular or intraventricular conduction abnormalities, and lead-specific QRS changes.	CD can alter ST/T morphology secondarily, which may create overlap with STTC decisions.
HYP	Hypertrophy-related ECG patterns.	Voltage criteria and associated repolarization or strain-type changes, often with lead-specific amplitude information.	HYP is less frequent and morphologically variable; lower F1 under external transfer should be interpreted with prevalence and threshold effects.

**Table 2:** Study cohorts and class-wise label counts.

Cohort	Records	NORM	MI	STTC	CD	HYP
PTB-XL eligible records	21,388	9514	5469	5235	4898	2649
PTB-XL test fold 10	2158	963	550	521	496	262
Ningbo external cohort	15,931	6299	880	6800	4688	637

**Table 3:** Cohort characteristics.

Characteristic	PTB-XL Eligible Cohort	Ningbo External Cohort
Number of ECG records	21,388	15,931
Role in study	Internal development/test	Frozen external validation
Age available, n (%)	21,103 (98.7%)	15,894 (99.8%)
Age missing, n (%)	285 (1.3%)	37 (0.2%)
Age, years, mean ± SD	59.40 ± 16.79	61.15 ± 19.09
Age, years, median (IQR)	61 (50–72)	64 (51–75)
Male, n (%)	11,111 (51.9%)	8643 (54.3%)
Female, n (%)	10,277 (48.1%)	7275 (45.7%)
Unknown sex, n (%)	0 (0.0%)	13 (0.1%)
Height available, n (%)	6913 (32.3%)	Not available
Height, cm, mean ± SD	166.79 ± 10.20	Not available
Height, cm, median (IQR)	166 (160–174)	Not available
Weight available, n (%)	9357 (43.7%)	Not available
Weight, kg, mean ± SD	71.08 ± 15.78	Not available
Weight, kg, median (IQR)	70 (60–80)	Not available
BMI available, n (%)	6686 (31.3%)	Not available
BMI, kg/m^2^, mean ± SD	25.24 ± 4.61	Not available
BMI, kg/m^2^, median (IQR)	24.78 (22.09–27.76)	Not available
Model input	12-lead ECG waveform only	12-lead ECG waveform only

**Table 4:** Preprocessing protocol.

Component	Implementation in This Study
Input duration and shape	10 s represented as 1000×12 samples for the five-superclass model.
Lead representation	Standard 12 clinical leads: I, II, III, aVR, aVL, aVF, and V1–V6.
Amplitude scaling	Record-wise, lead-wise z-score normalization using the mean and standard deviation of each lead within the same ECG.
Missing/non-finite values	Non-finite values were replaced by zero after normalization.
Feature engineering	No manual beat segmentation, QRS detector, handcrafted interval measurement, or externally fitted normalization statistics were used.
Internal/external consistency	The same preprocessing rule was applied to PTB-XL fold 10 and to Ningbo after the checkpoint and thresholds had already been fixed.
Clinical implication	Interpretability outputs can be read in ECG-domain units because the input remains a normalized 12-lead waveform rather than a transformed image or abstract feature vector.

**Table 5:** Model architecture.

Stage	Layer or Module	Main Configuration	Output Representation	Functional Role
Input	12-lead ECG signal	10 s recording at 100 Hz; lead-wise normalization	12×1000	Standardized multi-lead ECG input
Stem convolution	Conv1D + batch normalization + ReLU	32 filters, kernel size 7, same padding	32×1000	Initial extraction of local waveform morphology
InceptionTime block	Three stacked Inception modules	Parallel temporal kernels 9, 19, and 39 with pooling branch	128×1000	Multi-scale learning of QRS, ST/T, and broader waveform patterns
CNN refinement	Conv1D + batch normalization + ReLU + max pooling + dropout	160 filters followed by 192 filters	192×250	Feature refinement, channel expansion, and temporal compression before sequence modeling
Recurrent modeling	Bidirectional GRU	Hidden size 96 in each direction	250×192	Forward and backward temporal dependency learning within the 10 s ECG segment
Context encoding	Transformer encoder	Two encoder layers, 8 attention heads, feed-forward dimension 384, GELU activation	250×192	Longer-range temporal interaction modeling after convolutional downsampling
Temporal pooling	Attention pooling	Learnable weights over encoded time steps	192	Adaptive aggregation of diagnostically informative time regions
Output	Fully connected + sigmoid	Five independent diagnostic outputs	5 probabilities	Multi-label prediction for NORM, MI, STTC, CD, and HYP

**Table 6:** Model footprint and inference cost.

Batch Size	ms per Batch	ms per ECG	ECGs per Second
1	3.800	3.800	263
32	3.915	0.122	8173
128	12.405	0.097	10,318

Trainable parameters: 1,329,894; FP32 parameter storage: 5.07 MB. Timings exclude disk input/output and preprocessing.

**Table 7:** Validation-derived class-specific thresholds.

Class	NORM	MI	STTC	CD	HYP
Threshold	0.39	0.62	0.69	0.79	0.61

**Table 8:** Metric interpretation.

Metric	Question Answered	Why It Matters Clinically in This Study
AUROC	Does the model rank positive records above negative records across thresholds?	Useful for assessing discrimination under source shift, even when the fixed PTB-XL threshold is not optimal for Ningbo.
AUPRC	How well are positive cases retrieved when prevalence is imbalanced?	More informative than AUROC for minority labels such as HYP and externally uncommon labels such as MI in Ningbo.
Precision	Among records predicted positive, how many are truly positive?	Low precision means more false alarms and more clinician review burden.
Sensitivity/recall	Among true positive records, how many are detected?	Low sensitivity means missed target abnormalities, which is clinically important for screening.
Specificity	Among true negative records, how many are correctly rejected?	High specificity reduces false positives and unnecessary downstream review.
F1-score	Harmonic balance between precision and sensitivity at the fixed threshold.	Useful for summarizing thresholded decision quality, but should not be compared across papers unless labels, splits, thresholds, and prevalence are similar.
Exact match	Does the complete five-label vector match the reference vector?	Strict multi-label metric; penalizes any missed or extra superclass in the same ECG.
Hamming loss	What fraction of all record-class decisions is wrong?	Label-level error measure; 1 − Hamming loss is reported as label-wise accuracy for intuitive reading.
Macro average	What is the unweighted average across classes?	Highlights minority-class behavior and prevents frequent classes from dominating the conclusion.
Micro average	What is the pooled performance across all class decisions?	Summarizes overall decision behavior, but is influenced more by frequent labels.

**Table 9:** Internal class-wise performance.

Class	Positives	Threshold	AUROC	AUPRC	Precision	Sensitivity	Specificity	F1
NORM	963	0.39	0.9401	0.9139	0.7730	0.9408	0.7774	0.8487
MI	550	0.62	0.9242	0.8239	0.7468	0.7400	0.9142	0.7434
STTC	521	0.69	0.9324	0.8249	0.7049	0.8253	0.8900	0.7604
CD	496	0.79	0.9204	0.8379	0.7674	0.7319	0.9338	0.7492
HYP	262	0.61	0.8244	0.4698	0.3667	0.6298	0.8497	0.4635

**Table 10:** Internal decision-level error profile.

Class	TP	FP	FN	TN	Clinical Interpretation of the Main Error Pattern
NORM	906	266	57	929	High sensitivity for normal records, but false-positive NORM decisions can occur when abnormality evidence is weak or outside the five target labels.
MI	407	138	143	1470	False negatives remain clinically important because infarction-related morphology can be subtle or overlap with ST/T changes.
STTC	430	180	91	1457	High sensitivity with moderate false positives, consistent with broad repolarization patterns that may overlap with MI, HYP, or secondary CD changes.
CD	363	110	133	1552	High specificity suggests controlled false positives, while some conduction patterns remain missed at the fixed threshold.
HYP	165	285	97	1611	HYP remains the most difficult internal superclass; its low support and voltage/repolarization overlap produce low precision and F1.

**Table 11:** Exact-parent ablation.

Architecture	Params	Macro AUROC	Macro AUPRC	Micro-F1	Macro-F1	Exact Match	Hamming Loss
CNN-only	203,941	0.9113	0.7772	0.7608	0.7175	0.5709	0.1285
InceptionTime–CNN	568,806	0.9107	0.7704	0.7510	0.7140	0.5584	0.1374
InceptionTime–CNN–BiGRU	735,846	0.9095	0.7715	0.7537	0.7120	0.5626	0.1357
InceptionTime–CNN–Transformer	1,162,854	0.9080	0.7699	0.7485 *	0.7072	0.5431 *	0.1406 *
InceptionTime–CNN–BiGRU–Transformer	1,329,894	0.9080	0.7726	0.7624	0.7174	0.5649	0.1318

* Holm-adjusted p<0.05 versus the matched full architecture.

**Table 12:** Architecture sensitivity analysis.

Variant	Params	Macro AUROC	Macro AUPRC	Micro-F1	Macro-F1	Exact Match	Hamming Loss	Label-Wise acc.
CNN only	203,941	0.9142	0.7763	0.7696	0.7293	0.5746	0.1260	0.8740
CNN + BiGRU	370,981	0.9087	0.7682	0.7614	0.7177	0.5783	0.1296	0.8704
CNN + Transformer	845,989	0.9114	0.7816	0.7633	0.7214	0.5741	0.1302	0.8698
InceptionTime + CNN	934,981	0.9118	0.7753	0.7677	0.7251	0.5848	0.1237	0.8763
Post hoc full-stack sensitivity implementation	1,270,822	0.9122	0.7820	0.7702	0.7249	0.5931	0.1239	0.8761
InceptionTime + CNN + Transformer without BiGRU	1,282,374	0.9111	0.7710	0.7576	0.7148	0.5686	0.1329	0.8671
InceptionTime + CNN + Attention only	688,326	0.9112	0.7730	0.7631	0.7201	0.5871	0.1265	0.8735

**Table 13:** Paired primary-versus-CNN-only comparison.

Metric	Primary	CNN Only	Difference	95% CI	Raw *p*	Holm *p*
Macro AUROC	0.9083	0.9142	−0.0059	−0.0107 to −0.0011	0.0170	0.0390
Macro AUPRC	0.7741	0.7763	−0.0022	−0.0120 to 0.0079	0.6757	0.6757
Micro-F1	0.7517	0.7696	−0.0179	−0.0264 to −0.0087	0.0020	0.0100
Macro-F1	0.7130	0.7293	−0.0163	−0.0264 to −0.0050	0.0040	0.0160
Exact match	0.5524	0.5746	−0.0222	−0.0380 to −0.0051	0.0130	0.0390
Hamming loss	0.1390	0.1260	0.0130	0.0076 to 0.0179	0.0010	0.0060

**Table 14:** Internal and external performance.

Cohort	Macro AUROC	Macro AUPRC	Micro-F1	Macro-F1	Weighted-F1	Exact Match	Hamming Loss
PTB-XL fold 10	0.9083	0.7741	0.7517	0.7130	0.7577	0.5524	0.1390
Ningbo external	0.8885	0.6487	0.6507	0.5592	0.7169	0.4090	0.1968

**Table 15:** External class-wise performance.

Class	Positives	Threshold	AUROC	AUPRC	Precision	Sensitivity	Specificity	F1
NORM	6299	0.39	0.8867	0.8563	0.8287	0.7357	0.9005	0.7794
MI	880	0.62	0.9181	0.4441	0.1801	0.9148	0.7565	0.3009
STTC	6800	0.69	0.8462	0.7675	0.7131	0.7928	0.7625	0.7508
CD	4688	0.79	0.9060	0.8170	0.7614	0.6956	0.9091	0.7270
HYP	637	0.61	0.8854	0.3588	0.1396	0.8053	0.7933	0.2379

**Table 16:** Bootstrap confidence intervals.

Cohort	Metric	Estimate	Lower 95%	Upper 95%
PTB-XL fold 10	Macro AUROC	0.9083	0.8998	0.9164
	Macro AUPRC	0.7741	0.7565	0.7912
	Micro-F1	0.7517	0.7392	0.7640
	Macro-F1	0.7130	0.6987	0.7272
	Exact match	0.5524	0.5315	0.5737
	Hamming loss	0.1390	0.1316	0.1464
Ningbo external	Macro AUROC	0.8885	0.8847	0.8923
	Macro AUPRC	0.6487	0.6383	0.6601
	Micro-F1	0.6507	0.6458	0.6556
	Macro-F1	0.5592	0.5538	0.5648
	Exact match	0.4090	0.4015	0.4164
	Hamming loss	0.1968	0.1938	0.1999

**Table 17:** Class-wise calibration.

Cohort	Class	ECE	Brier Score
PTB-XL fold 10	NORM	0.0473	0.1005
	MI	0.0795	0.1039
	STTC	0.1192	0.1164
	CD	0.1590	0.1234
	HYP	0.2158	0.1587
	**Macro**	**0.1241**	**0.1206**
Ningbo external	NORM	0.0845	0.1325
	MI	0.3287	0.2156
	STTC	0.1377	0.1798
	CD	0.1573	0.1428
	HYP	0.3394	0.1970
	**Macro**	**0.2095**	**0.1735**

Bold values indicate macro-averaged results.

**Table 18:** Age- and sex-stratified performance.

Cohort	Subgroup	N	Macro AUROC	Macro AUPRC	Micro-F1	Macro-F1	Exact Match	Hamming Loss
PTB-XL	Overall	2158	0.9083	0.7741	0.7517	0.7130	0.5524	0.1390
PTB-XL	Male	1048	0.8971	0.7631	0.7311	0.6924	0.5401	0.1498
PTB-XL	Female	1110	0.9187	0.7862	0.7711	0.7321	0.5640	0.1288
PTB-XL	Age <60	923	0.9050	0.7259	0.7984	0.6805	0.6880	0.0969
PTB-XL	Age ≥60	1203	0.8927	0.7726	0.7214	0.7066	0.4489	0.1714
Ningbo	Overall	15,931	0.8885	0.6487	0.6507	0.5592	0.4090	0.1968
Ningbo	Male	8643	0.8695	0.6336	0.6237	0.5509	0.3486	0.2202
Ningbo	Female	7275	0.9065	0.6481	0.6859	0.5549	0.4811	0.1689
Ningbo	Age <60	6339	0.9024	0.6442	0.7027	0.5305	0.5633	0.1463
Ningbo	Age ≥60	9555	0.8625	0.6323	0.6233	0.5501	0.3073	0.2301

**Table 19:** Lead-occlusion effects.

Class	Lead	Mean Probability Drop
NORM	V3	0.092
NORM	aVF	0.091
NORM	V2	0.086
MI	III	0.047
MI	I	0.034
MI	aVL	0.024
STTC	V1	0.043
STTC	aVF	0.028
STTC	III	0.021
CD	V1	0.044
CD	II	0.039
CD	V3	0.031
HYP	V1	0.064
HYP	V6	0.039
HYP	V5	0.022

**Table 20:** Comparison with recent multi-label ECG studies.

Study	Task/Data	Evaluation Design	Reported Headline Result	Direct-Comparison Constraint
Strodthoff et al. (2021) [9]	PTB-XL diagnostic benchmarking	Standardized internal benchmark	Model-dependent	Benchmark scope and outputs differ
Zhou and Chen (2024) [30]	Lead-wise multi-label ECG	Internal multi-label evaluation	NR here	Different architecture and reporting protocol
Zeng et al. (2026) [27]	Interpretable PTB-XL multi-label ECG	Internal attention, SHAP, and Grad-CAM study	NR here	No frozen external Ningbo evaluation reported
Al-Bairmani et al. (2026) [33]	27-label Challenge 2020 ECG	Mixed-source task; compact model	Accuracy 0.9124; macro-F1 0.8301	Different labels, sources, split, and metric definitions
Present study	Five PTB-XL superclasses	Official folds; frozen Ningbo evaluation	Internal/external macro-AUROC 0.9083/0.8885	Cross-study numerical ranking remains inappropriate

**Figure A1:**
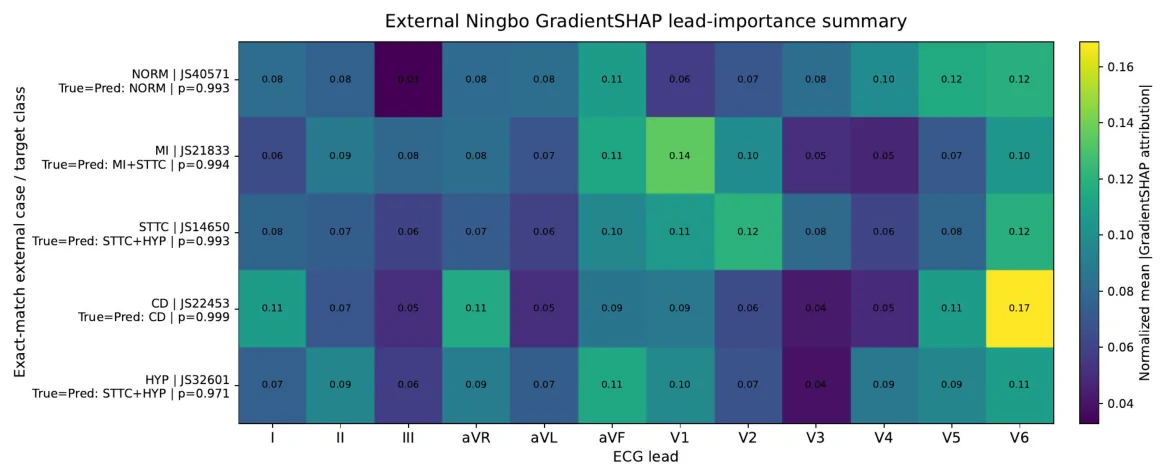
GradientSHAP lead attributions.

## Data Availability

PTB-XL and the Ningbo ECG resources are publicly available through PhysioNet (https://physionet.org/) subject to the original repository terms.

## Abbreviations

The following abbreviations are used in this manuscript:

AI	Artificial intelligence
AUPRC	Area under the precision–recall curve
AUROC	Area under the receiver operating characteristic curve
CD	Conduction disturbance
ECG	Electrocardiogram
ECE	Expected calibration error
HYP	Hypertrophy
MI	Myocardial infarction
NORM	Normal ECG
STTC	ST/T change

## Appendix A. Supplementary SHAP-Style Lead-Attribution Analysis

As an additional SHAP-style explanation, we applied GradientSHAP to the same representative exact-match Ningbo cases used for the external Grad-CAM analysis. GradientSHAP provides an efficient gradient-based approximation to SHAP-style attribution for deep neural networks and is computationally more practical than exact KernelSHAP for 12-lead ECG signals. This analysis complements temporal Grad-CAM by summarizing lead-level contribution patterns across the five target superclasses. These attributions describe selected individual cases; they are not a cohort-level summary and were not used to validate the mean lead-occlusion effects reported in Table 19. Figure A1 shows the supplementary GradientSHAP lead-attribution heatmap.

## References

[1] Kligfield P., Gettes L.S., Bailey J.J., Childers R., Deal B.J., Hancock E.W., van Herpen G., Kors J.A., Macfarlane P., Mirvis D.M. (2007). Recommendations for the standardization and interpretation of the electrocardiogram: Part I: The electrocardiogram and its technology: A scientific statement from the American Heart Association Electrocardiography and Arrhythmias Committee, Council on Clinical Cardiology; the American College of Cardiology Foundation; and the Heart Rhythm Society: Endorsed by the International Society for Computerized Electrocardiology. Circulation.

[2] Surawicz B., Childers R., Deal B.J., Gettes L.S. (2009). AHA/ACCF/HRS recommendations for the standardization and interpretation of the electrocardiogram: Part III: Intraventricular conduction disturbances: A scientific statement from the American Heart Association Electrocardiography and Arrhythmias Committee, Council on Clinical Cardiology; the American College of Cardiology Foundation; and the Heart Rhythm Society: Endorsed by the International Society for Computerized Electrocardiology. J. Am. Coll. Cardiol..

[3] Zhang M.-L., Zhou Z.-H. (2014). A review on multi-label learning algorithms. IEEE Trans. Knowl. Data Eng..

[4] Cabitza F., Campagner A. (2021). The need to separate the wheat from the chaff in medical informatics: Introducing a comprehensive checklist for the (self)-assessment of medical AI studies. Int. J. Med. Inform..

[5] Marconi L., Cabitza F. (2025). Show and tell: A critical review on robustness and uncertainty for a more responsible medical AI. Int. J. Med. Inform..

[6] Hannun A.Y., Rajpurkar P., Haghpanahi M., Tison G.H., Bourn C., Turakhia M.P., Ng A.Y. (2019). Cardiologist-level arrhythmia detection and classification in ambulatory electrocardiograms using a deep neural network. Nat. Med..

[7] Ribeiro A.H., Ribeiro M.H., Paixão G.M.M., Oliveira D.M., Gomes P.R., Canazart J.A., Ferreira M.P.S., Andersson C.R., Macfarlane P.W., Meira W. (2020). Automatic diagnosis of the 12-lead ECG using a deep neural network. Nat. Commun..

[8] Attia Z.I., Kapa S., López-Jiménez F., McKie P.M., Ladewig D.J., Satam G., Pellikka P.A., Enriquez-Sarano M., Noseworthy P.A., Munger T.M. (2019). Screening for cardiac contractile dysfunction using an artificial intelligence-enabled electrocardiogram. Nat. Med..

[9] Strodthoff N., Wagner P., Schaeffter T., Samek W. (2021). Deep learning for ECG analysis: Benchmarks and insights from PTB-XL. IEEE J. Biomed. Health Inform..

[10] Fawaz H.I., Forestier G., Weber J., Idoumghar L., Muller P.-A. (2019). Deep learning for time series classification: A review. Data Min. Knowl. Discov..

[11] Fawaz H.I., Lucas B., Forestier G., Pelletier C., Schmidt D.F., Weber J., Webb G.I., Idoumghar L., Muller P.-A., Petitjean F. (2020). InceptionTime: Finding AlexNet for time series classification. Data Min. Knowl. Discov..

[12] Hochreiter S., Schmidhuber J. (1997). Long short-term memory. Neural Comput..

[13] Cho K., van Merriënboer B., Gülçehre C., Bahdanau D., Bougares F., Schwenk H., Bengio Y. (2014). Learning phrase representations using RNN encoder-decoder for statistical machine translation. Proceedings of the 2014 Conference on Empirical Methods in Natural Language Processing (EMNLP).

[14] Bahdanau D., Cho K., Bengio Y. Neural machine translation by jointly learning to align and translate. Proceedings of the 3rd International Conference on Learning Representations (ICLR).

[15] Vaswani A., Shazeer N., Parmar N., Uszkoreit J., Jones L., Gomez A.N., Kaiser L., Polosukhin I. (2017). Attention is all you need. Advances in Neural Information Processing Systems.

[16] He K., Zhang X., Ren S., Sun J. Deep residual learning for image recognition. Proceedings of the IEEE Conference on Computer Vision and Pattern Recognition.

[17] Wagner P., Strodthoff N., Bousseljot R.-D., Kreiseler D., Lunze F.I., Samek W., Schaeffter T. (2020). PTB-XL, a large publicly available electrocardiography dataset. Sci. Data.

[18] Wagner P., Strodthoff N., Bousseljot R.-D., Samek W., Schaeffter T. (2022). PTB-XL, a large publicly available electrocardiography dataset (version 1.0.3). PhysioNet.

[19] Goldberger A.L., Amaral L.A.N., Glass L., Hausdorff J.M., Ivanov P.C., Mark R.G., Mietus G.B., Moody G.B., Peng C.-K., Stanley H.E. (2000). PhysioBank, PhysioToolkit, and PhysioNet: Components of a new research resource for complex physiologic signals. Circulation.

[20] Perez Alday E.A., Gu A., Shah A.J., Robichaux C., Wong A.K.I., Liu C., Liu F., Rad A.B., Elola A., Seyedi S. (2021). Classification of 12-lead ECGs: The PhysioNet/Computing in Cardiology Challenge 2020. Physiol. Meas..

[21] Reyna M., Sadr N., Gu A., Perez Alday E.A., Liu C., Seyedi S., Shah A., Clifford G.D. (2022). Will Two Do? Varying dimensions in electrocardiography: The PhysioNet/Computing in Cardiology Challenge 2021 (version 1.0.3). PhysioNet.

[22] Leinonen T., Wong D., Vasankari A., Wahab A., Nadarajah R., Kaisti M., Airola A. (2024). Empirical investigation of multi-source cross-validation in clinical ECG classification. Comput. Biol. Med..

[23] Ribeiro M.T., Singh S., Guestrin C. “Why should I trust you?”: Explaining the predictions of any classifier. Proceedings of the KDD ’16: The 22nd ACM SIGKDD International Conference on Knowledge Discovery and Data Mining.

[24] Lundberg S.M., Lee S.-I. (2017). A unified approach to interpreting model predictions. Advances in Neural Information Processing Systems.

[25] Selvaraju R.R., Cogswell M., Das A., Vedantam R., Parikh D., Batra D. Grad-CAM: Visual explanations from deep networks via gradient-based localization. Proceedings of the IEEE International Conference on Computer Vision.

[26] Samek W., Wiegand T., Müller K.-R. (2017). Explainable artificial intelligence: Understanding, visualizing and interpreting deep learning models. arXiv.

[27] Zeng W., Shan L., Chen Y., Ma Y., Du S. (2026). Interpretable deep learning framework for multi-label ECG classification: Enhancing cardiac diagnostics through feature attention and explainability. Biomed. Signal Process. Control.

[28] Che C., Zhang P., Zhu M., Qu Y., Jin B. (2021). Constrained transformer network for ECG signal processing and arrhythmia classification. BMC Med. Inform. Decis. Mak..

[29] Bai X., Dong X., Li Y., Liu R., Zhang H. (2024). A hybrid deep learning network for automatic diagnosis of cardiac arrhythmia based on 12-lead ECG. Sci. Rep..

[30] Zhou F., Chen L. (2024). Leadwise clustering multi-branch network for multi-label ECG classification. Med. Eng. Phys..

[31] Goettling M., Hammer A., Malberg H., Schmidt M. (2024). xECGArch: A trustworthy deep learning architecture for interpretable ECG analysis considering short-term and long-term features. Sci. Rep..

[32] Zhou X., Li T., Hayama H., Nakamura K., Liu S.-H., Chen W., Zhu X. (2025). Diagnosis of cardiac conditions from 12-lead electrocardiogram through natural language supervision. npj Digit. Med..

[33] Al-Bairmani M.T., Yazdchi M., Nasimi F. (2026). A joint CNN-Bi-LSTM-transformer architecture with SHAP explanations for multi-label arrhythmia detection from 12-lead ECGs. Sci. Rep..

[34] Zheng J., Zhang J., Danioko S., Yao H., Guo H., Rakovski C. (2020). A 12-lead electrocardiogram database for arrhythmia research covering more than 10,000 patients. Sci. Data.

[35] Zheng J., Guo H., Chu H. (2022). A large scale 12-lead electrocardiogram database for arrhythmia study (version 1.0.0). PhysioNet.

[36] Reyna M.A., Sadr N., Perez Alday E.A., Gu A., Shah A.J., Robichaux C., Rad A.B., Elola A., Seyedi S., Ansari S. (2022). Issues in the automated classification of multilead ECGs using heterogeneous labels and populations. Physiol. Meas..

[37] Ridnik T., Ben-Baruch E., Zamir N., Noy A., Friedman I., Protter M., Zelnik-Manor L. Asymmetric loss for multi-label classification. Proceedings of the IEEE/CVF International Conference on Computer Vision.

[38] Collins G.S., Moons K.G.M., Dhiman P., Riley R.D., Beam A.L., Calster B.V., Ghassemi M., Liu X., Reitsma J.B., van Smeden M. (2024). TRIPOD+AI statement: Updated guidance for reporting clinical prediction models that use regression or machine learning methods. BMJ.

[39] Van Calster B., McLernon D.J., van Smeden M., Wynants L., Steyerberg E.W. (2019). Calibration: The Achilles heel of predictive analytics. BMC Med..

[40] Liu X., Cruz Rivera S., Moher D., Calvert M.J., Denniston A.K., SPIRIT-AI and CONSORT-AI Working Group (2020). Reporting guidelines for clinical trial reports for interventions involving artificial intelligence: The CONSORT-AI extension. Nat. Med..

[41] Kingma D.P., Ba J. Adam: A method for stochastic optimization. Proceedings of the 3rd International Conference on Learning Representations (ICLR).

[42] Loshchilov I., Hutter F. Decoupled weight decay regularization. Proceedings of the 7th International Conference on Learning Representations (ICLR).

[43] Saito T., Rehmsmeier M. (2015). The precision–recall plot is more informative than the ROC plot when evaluating binary classifiers on imbalanced datasets. PLoS ONE.

